# Covalent Organic Frameworks in Smart Food Packaging: Advances, Challenges, and Future Prospects

**DOI:** 10.1111/1541-4337.70662

**Published:** 2026-09-29

**Authors:** Sara Esteghlal, Hossein Daneshgar, Navid Rabiee, Mohammadreza Khalesi

**Affiliations:** ^1^ Department of Food Science and Technology, School of Agriculture Shiraz University Shiraz Iran; ^2^ Department of Physics Sharif University of Technology Tehran Iran; ^3^ Department of Biomaterials, Saveetha Dental College and Hospitals, SIMATS Saveetha University Chennai India; ^4^ Department of Biological Sciences University of Limerick Limerick Ireland; ^5^ Bernal Institute University of Limerick Limerick Ireland; ^6^ Health Research Institute University of Limerick Limerick Ireland

**Keywords:** covalent organic framework (COF), food packaging, sustainable materials, sustainable packaging

## Abstract

Food preservation challenges have positioned packaging as a rapidly evolving, innovation‐driven field, with smart packaging offering transformative solutions. Among advanced materials, covalent organic frameworks (COFs) have attracted growing attention due to their permanent porosity, structural tunability, high surface area, and chemical stability. These features allow COFs to address critical needs in food packaging, including active barrier protection, antimicrobial action, and smart sensing of food freshness and spoilage. This review examines recent progress in applying COFs to food packaging, with particular focus on their ability to prolong shelf life and enable smart functionalities for real‐time monitoring of food quality. Safety aspects, biocompatibility, recyclability, and biodegradability are also critically evaluated, as these remain key considerations for regulatory approval and industrial translation. Although current studies report minimal cytotoxicity, long‐term toxicological assessments are still limited, underscoring the need for more comprehensive investigations. Looking forward, artificial intelligence (AI) and machine learning (ML) provide powerful tools for accelerating COF discovery and optimization. Their integration into COF design could streamline synthesis, improve scalability, and guide application‐specific material development. COFs represent a promising new class of materials poised to advance the next generation of sustainable, functional, and smart food‐packaging systems.

## 1 Introduction

Packaging of food products is of great importance due to its role in maintaining the food quality and protecting it against environmental damages like physical and thermal damages as well as moisture and oxygen, as the main reasons for degradation, oxidation, and microbial deterioration of foods during the distribution chain and storage (Jafarzadeh et al. 2023). Global food systems face unprecedented challenges from climate change, population growth, and resource constraints, with approximately one‐third of all produced food lost or wasted annually, representing 1.3 billion tons of squandered resources and 8% of global greenhouse gas emissions (Aslam et al. 2024). Food packaging, positioned at the critical interface between production and consumption, offers a strategic intervention point for addressing these challenges through materials innovation. Petroleum‐based materials have a widespread application in food packaging due to appropriate mechanical and barrier properties, low price, flexibility, and versatility. However, due to their negative environmental and health impact, seeking new biodegradable and environmentally friendly materials, such as organic materials and biopolymers for food packaging, seems to be crucial (Alizadeh Sani et al. 2024; Esteghlal et al. 2016). Accordingly, there is an increasing interest in the development of innovative and sustainable packaging materials that benefit from new functional properties (Alizadeh Sani et al. 2024). Using smart food packaging in this regard can enhance the shelf life of food products and decrease environmental damage at the same time. Incorporation of nanomaterials into biodegradable and eco‐friendly food‐packaging materials can improve their performance in food protection and enhance food quality and safety. Among various types of nanomaterials, porous nanomaterials have recently gained considerable attention due to their unique properties (Jafarzadeh et al. 2023). Within this context, the replacement of conventional plastics with bio‐based and biodegradable polymers has emerged as a promising pathway toward sustainable packaging (RameshKumar et al. 2020; Imre and Pukánszky 2013). Natural polymers extracted from renewable resources such as cellulose, lignin, starch, and chitin, as well as biopolymers biosynthesized by microorganisms (e.g., polyhydroxyalkanoates) or synthesized from biomass‐derived monomers (e.g., polylactic acid), represent key candidates for the next generation of food‐packaging materials (Garrison et al. 2016; Oliver‐Cuenca et al. 2024). These bio‐derived building blocks not only reduce dependence on fossil resources but also align with global sustainability goals by offering controlled degradation pathways and, in some cases, superior mechanical and barrier properties compared with petroleum‐derived plastics (Lambert and Wagner 2017; Okolie et al. 2023). However, their widespread adoption is constrained by challenges such as batch‐to‐batch variability, insufficient scalability of raw material supply, and incomplete understanding of long‐term environmental impacts. The transition from laboratory‐scale bio‐based materials to commercial applications faces several critical challenges that must be addressed systematically (Figure 1) (Tardy et al. 2023). Recent research has therefore moved toward hybrid approaches that integrate bio‐based polymers with advanced functional nanomaterials, particularly porous frameworks, to overcome these limitations. Porous nanomaterials, including covalent organic frameworks (COFs) and hydrogen‐bonded covalent organic polymers (HCOPs), offer high surface area, tunable pore structures, and chemical stability, enabling their use as active components in smart food packaging (Han et al. 2023; Stoica et al. 2024; Qiu et al. 2025). By enhancing gas selectivity, modulating water vapor permeability, and providing antimicrobial and antioxidant functions, these materials can extend shelf life while simultaneously reducing environmental burden. Consequently, the synergy between biodegradable polymers and porous nanomaterials is reshaping the design principles of packaging, offering a pathway toward intelligent, sustainable, and high‐performance solutions for global food preservation.

**FIGURE 1:**
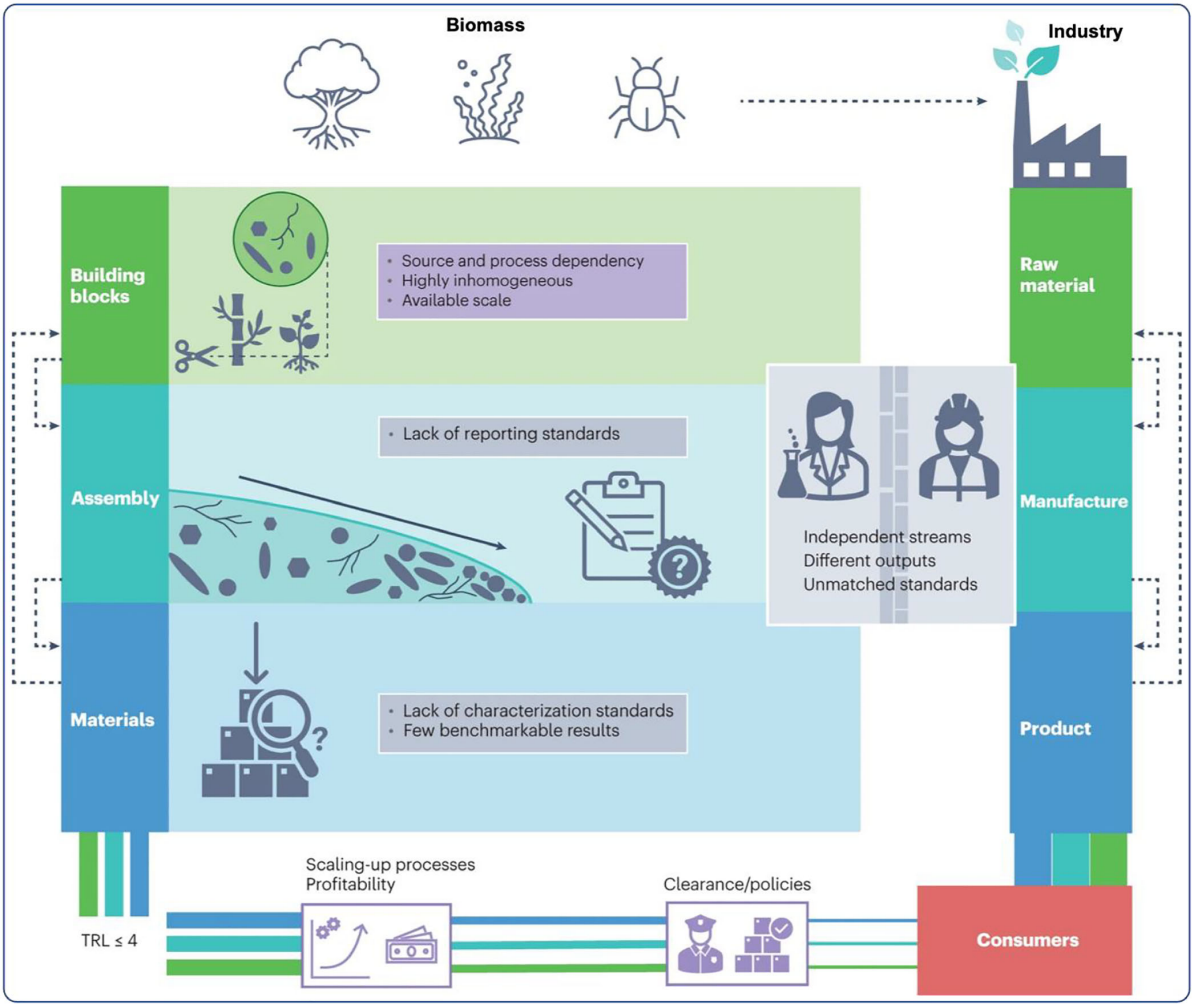
The upper section illustrates symbolic depictions of biomass resources, predominantly lignocellulosic materials derived from plants and trees, emphasizing their prospective role in sustainable processing pathways. The figure outlines three major developmental directions: (1) extraction of fundamental components, (2) their subsequent assembly, and (3) the generation of end‐use materials. These activities are framed within parallel academic initiatives (left) and industrial efforts (right). At the bottom, the diagram traces the transition from laboratory research to consumer products, marking the technological readiness level (TRL) that delineates this progression. Key challenges intrinsic to bio‐based approaches are highlighted in purple, whereas additional barriers that must be addressed to accelerate translation are presented in gray (Tardy et al. 2023). Reprinted with permission from Tardy et al. (2023), Copyright 2023, Springer Nature.

Traditional packaging approaches, while effective at basic protection, lack the intelligence and responsiveness needed to address these complex challenges, driving innovation toward smart, adaptive materials. Porous materials, characterized by their regular arrays of void spaces and exceptional surface‐to‐volume ratios, operate through sophisticated host–guest interactions, where carefully engineered pore environments can selectively capture, detect, or release target molecules with remarkable specificity (Arghavani et al. 2024). Recent advances in synthetic chemistry have expanded the porous materials landscape dramatically, from traditional inorganic zeolites to metal–organic frameworks (MOFs) and, most recently, to entirely organic porous networks with adjustable structure and better compatibility. The application potential of these materials extends across three principal functional domains in food packaging: (1) selective adsorption systems that trap undesirable compounds such as ethylene, oxygen, moisture, or microbial products; (2) sensing platforms that detect and signal changes in food quality through colorimetric, fluorescent, or electronic responses; and (3) controlled release systems that deliver antimicrobial, antioxidant, or other functional agents in response to environmental triggers. These capabilities, already demonstrated in laboratory settings, promise to transform conventional packaging into responsive carriers capable of extending shelf life, signaling freshness, and enhancing safety through molecularly precise interactions (Zaitoon et al. 2022). The extraordinary potential of porous materials for food packaging remains partially constrained; however, challenges in scalable synthesis, cost‐effective production, and demonstration of long‐term safety for food contact applications still exist (Jafarzadeh et al. 2023; Kim et al. 2022). The wide variety of these materials offers many design options but also makes it difficult to choose the best one. It requires careful methods to explore this complex field effectively. Combining porous materials science with artificial intelligence (AI) and data science can speed up the discovery of new materials, helping design ones that are specifically tailored to solve food preservation problems. This review examines the potential of COFs, an emerging class of porous, lightweight crystalline materials, for advanced food‐packaging applications, highlighting their unique structural attributes, functional capabilities, and potential ability to improve food preservation and safety. Therefore, the primary aim of this review is to provide a comprehensive and critical evaluation of COFs as emerging functional materials for smart food packaging. Specifically, this work focuses on (1) elucidating the fundamental structural and physicochemical properties of COFs relevant to food preservation and monitoring; (2) analyzing their functional mechanisms in enhancing barrier performance, antimicrobial activity, and sensing capability; (3) evaluating their safety, sustainability, and regulatory aspects; and (4) identifying future research directions, including the role of AI in accelerating COF discovery and industrial translation.

## 2 Porous Materials in Advanced Applications

Porous materials are materials with void spaces and pores, which makes them an appropriate tool for the adsorption of guest molecules through physical or chemical mechanisms. The extensive internal surface area generated by the pore network can substantially enhance the adsorption capacity of porous materials. Considering the surface chemistry of porous materials, a suitable host can be designed and fabricated for each group of guest molecules for certain applications. There are various types of porous materials with a wide range of structures and features. Pore size and morphology, degree of porosity, crystallinity or amorphousness, thermal durability, surface charge, chemistry, and modification, particle size, and surface‐to‐volume ratio are the important characteristics of porous materials that affect their functionality (Arghavani et al. 2025).

Porous materials can be classified on the basis of various parameters like their pore size, structure, material type, intended applications, and so forth. On the basis of the pore size, solid porous materials are classified as microporous with a pore size of <2 nm, mesoporous with a pore size of 2–50 nm, and macroporous materials with pores larger than 50 nm. The pore size and shape of the pores directly affect the functionality of porous materials (Davis 2002). They can also be categorized as porous materials with ordered structure, possessing a regular, repeating lattice structure, or amorphous or disordered materials, without long‐range order or irregular pores. Although ordered porous materials have been the focus and are supposed to be vital in engineering different characteristics, there is increasing evidence on the importance of disordered structures in developing novel characteristics (Bennett et al. 2021). On the basis of another categorization, they fall into three main groups: organic, inorganic, and hybrid porous materials, each with further detailed subcategories. Among the most prominent inorganic porous materials are zeolites, silica, alumina, and clays. These materials exhibit exceptional resistance to environmental stressors such as heat, making them highly attractive for applications in industries that operate under harsh conditions. On the organic side, materials, such as COFs and porous polymers, are considered key representatives, offering tunable surface chemistry and lower density. Hybrid materials, like MOFs and PCPs, on the other hand, exhibit even greater diversity due to the flexibility of their design, combining the stability of inorganic clusters with the tunability of organic linkers (Hüsing 2006). Different classifications of porous materials with some examples of each category can be observed in Figure 2.

**FIGURE 2:**
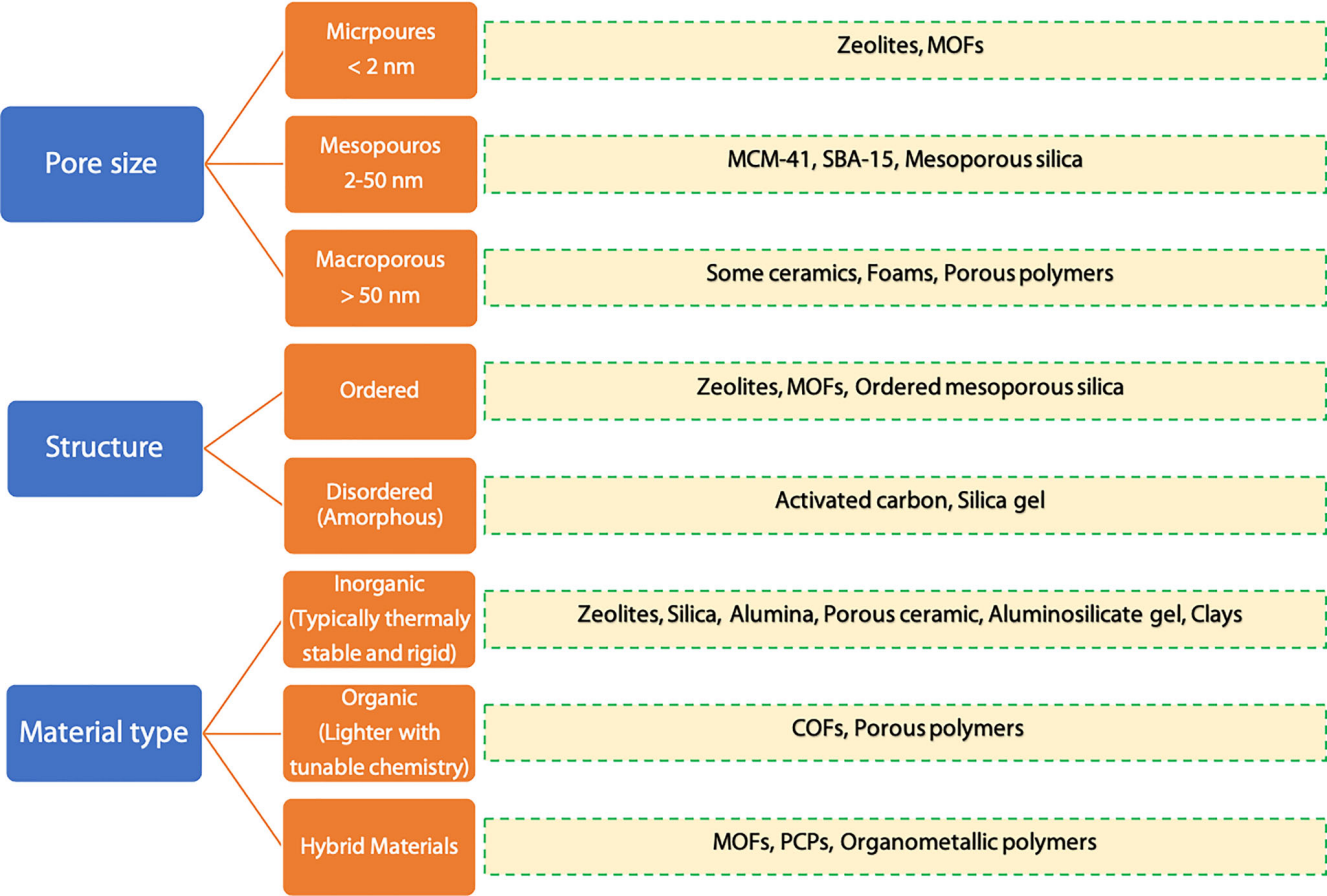
Schematic illustration of different classifications of porous materials. COFs: covalent organic frameworks; MOFs: metal–organic frameworks; PCP: porous coordination polymers.

### 2.1 Role of Porous Materials in Food Packaging

Due to the unique properties of porous materials, they are materials of interest to be incorporated into food‐packaging materials and enhance the quality and shelf life of the packed foods. They have a high surface‐to‐volume ratio, high mechanical and thermal durability, and controllable pore size, particle size, and chemical structure, which can be tailored on the basis of the desired application in food packaging. Additionally, some of them with bactericide impact or the ability to adsorb antimicrobial compounds are preferred to enhance food shelf life. These properties affect the interactions of porous materials and food‐packaging materials and subsequently, their functional efficiency (Jafarzadeh et al. 2023). Although porous materials can offer many advantages to food packaging, their level of safety, nontoxicity, and ideally edibility are the most important parameters to consider before application in the food packaging (Moussa et al. 2016).

Among the various benefits of incorporation of porous materials into food‐packaging materials, imposing antimicrobial, antioxidant, and gas adsorption ability through encapsulation/adsorption of bioactive agents, controlling the ripening process of fruits and vegetables via the controlled release of ripening compounds, improving the barrier properties of the packaging films against moist, light, oxygen, and so forth, enhancing the thermal and mechanical durability, and introducing stimuli‐responsive features can be mentioned. Reaching the maximum efficiency in food packaging, porous materials need to have a high selectivity for bioactives. For encapsulation‐based applications, appropriate affinity and selectivity toward bioactive compounds are important for achieving effective loading and controlled release. The ability of porous materials to encapsulate and then sustain release of bioactive compounds results in more efficient functionality due to the protective impact of the capsule (porous material) against the environmental conditions and longer exposure time of the food to the bioactives without affecting taste and odor (Azizi‐Lalabadi and Jafari 2021; Sullivan et al. 2021). In addition to the bioactive compounds, fluorescent dyes can be encapsulated in the porous materials as sensors for food pollutants like veterinary drugs, heavy metals, and illegal ingredients (like certain artificial colors [red No. 40 and yellow Nos. 4 and 6], potassium bromide, brominated vegetable oil, and parabens) or mycotoxins and pathogens (Jafarzadeh et al. 2023).

### 2.2 Types of Porous Materials Used in Food Packaging

The selection of an appropriate porous material for food packaging hinges on a thorough understanding of the intrinsic properties and functionalities of the candidate materials (Lee et al. 2019; Udenni Gunathilake et al. 2017). Each porous material exhibits unique structural, chemical, and physical characteristics that directly influence its suitability for specific packaging applications, such as gas adsorption, moisture regulation, antimicrobial activity, and controlled release of preservatives or antioxidants (Kitagawa 2017; Slater and Cooper 2015; Sing 2004). According to the literature, several classes of porous materials have emerged as prominent candidates for advanced food‐packaging systems. MOFs are highly valued for their tunable pore sizes, exceptional surface areas, and ability to selectively adsorb gases or volatile compounds. Zeolites, with their crystalline microporous structures, offer excellent molecular sieving and stability under various environmental conditions. Mesoporous silica nanoparticles (MSNs) combine high surface area with chemical versatility, enabling the encapsulation and controlled release of bioactive agents. Additionally, carbon‐based nanomaterials, including graphene derivatives and activated carbons, provide outstanding mechanical strength, electrical conductivity, and adsorption capabilities (Davis 2002; Lu et al. 1999; Bennett et al. 2021; Qin et al. 2016). A strategic choice among these porous materials, tailored to the specific functional requirements of the packaging, is critical for optimizing food preservation, safety, and shelf life.

#### 2.2.1 Metal–Organic Framework

MOFs are a class of porous hybrid materials constructed from metal ions, often including transition or alkaline earth metals, coordinated with organic linkers to form crystalline or, in some cases, amorphous frameworks with stable, permanent porosity. By varying the metal nodes, organic linkers, and reaction conditions, an extensive diversity of MOF architectures can be achieved, offering highly tunable pore sizes, channel geometries, and surface functionalities. These materials exhibit large specific surface areas, adjustable physicochemical properties, and easily modifiable surfaces, making them exceptionally versatile. Such characteristics have enabled a broad range of applications, including gas storage and separation, selective adsorption of molecules, biosensing and pathogen detection, targeted delivery of therapeutics, and antimicrobial functionality, particularly in the context of food packaging. The combination of structural tunability and functional versatility positions MOFs as promising candidates for designing advanced, high‐performance, and multifunctional materials in both industrial and biomedical applications (Yao et al. 2024).

#### 2.2.2 Zeolite

Zeolites are a class of inorganic crystalline microporous materials built from TO_4_ tetrahedral units, where T typically represents aluminum, silicon, or phosphorus. The inherent negative charge of these tetrahedra is balanced by extra‐framework cations, which occupy positions within the pore network. The combination of oxygen atoms and compensating cations generates a high adsorption capacity, making zeolites effective for selective capture and separation processes. These materials are distinguished by their exceptional thermal and chemical stability, uniform pore dimensions, cost‐effectiveness, and nontoxic nature. Zeolites are available in both natural and synthetic forms, offering versatility for applications ranging from catalysis and ion exchange to adsorption and environmental remediation. The first synthetic zeolite, aluminosilicate mordenite, was introduced in 1948 (Li and Yu 2021). Zeolite A, zeolite Y, zeolite Socony Mobil‐5 (Wattanawong et al. 2020), zeolite imidazole (Alizadeh Sani et al. 2024), zeolite A4 (Dogan et al. 2009), and clinoptilolite (do Nascimento Sousa et al. 2020) are examples of synthetic and natural zeolites used in food packaging.

#### 2.2.3 Mesoporous Silica Nanoparticles

Mesoporous silica materials are inorganic ordered materials with high pore volume. Beyond large surface area, controllable pore size and dimension, and ease of surface modification, they exhibit excellent colloidal stability and high dispersibility. Following the discovery of M41S, a group of molecular sieves, MSNs became a focal point in material research. The most widely used silica mesoporous materials are MCM‐41, MCM‐48, and SBA‐15 that exhibit 2D‐hexagonal and three‐dimensional (3D)‐cubic structures. Mesoporous silica materials do not have any toxicity at an appropriate amount as a result of the abundance of silanol groups on the surface and are easily internalized by the cells, which makes them an appropriate, safe porous material (Kankala et al. 2020; Manzano and Vallet‐Regí 2020; Mehmood et al. 2017) for food‐packaging applications.

#### 2.2.4 Carbon‐Based Nanomaterials

Carbon‐based nanomaterials are a wide range of materials with a crystallographic framework based on carbon atoms with an intricate internal pore architecture. The diversity in the binding of carbon atoms leads to different forms of allotropes and subsequently, various types of carbon‐based nanomaterials such as carbon dots, graphene dots, carbon nanotubes, and activated carbon that have been used in food‐packaging research. Due to the large surface area and porosity, the presence of various functional groups on their surface, and desired optical and mechanical properties, carbon‐based nanomaterials have a wide potential for applications (Díez‐Pascual 2021). Considering their green fabrication process and bactericide effect, they are interesting porous materials for application in food packaging (Raul et al. 2022). Various allotropic and structural forms of carbon impact the colonization behavior and biological responses of foodborne pathogens. The impact of carbon‐based nanomaterials on live organisms is strongly based on their allotropic structure. For instance, graphene has been reported to cause damage to cells and cell membranes, whereas nanodiamonds have been reported to exhibit pathogen‐deactivation activity with comparatively limited adverse effects on cell membranes and genetic material in the cited study. These findings suggest that nanodiamonds may warrant further investigation for food‐packaging applications; however, their safety requires comprehensive evaluation before practical food‐contact applications (Mitura et al. 2021).

### 2.3 Limitations of Using Porous Materials in Food Packaging

As mentioned, zeolite, MOF, MSNs, and carbon‐based nanomaterials are the porous materials with the highest application in food packaging (Jafarzadeh et al. 2023). However, due to some limitations, seeking new porous materials is still ongoing. Although zeolites and carbon‐based nanomaterials are inorganic porous materials, scientists are increasingly focusing on organic porous materials due to their superior properties, and their advantages over inorganic ones, such as better controllability of the structure and functionality of the organic porous materials, as well as flexibility in tailoring their features for targeted applications. Considering these, various organic materials, such as porous polymer networks, polymers of intrinsic microporosity, and conjugated microporous polymers, have been fabricated recently. These organic materials exhibit amorphous structures with multifunctional units that are bonded via covalent interactions. Owing to their amorphousness, they exhibit a wide range of pore sizes, which prevents their applications for certain goals. MOFs, as a group of hybrid porous materials with a crystalline structure, show high structural adjustability, homogeneous pores, and high surface area, features that make them the center of research interest. However, some types of MOFs contain metal ions with toxicity effects and high reactivity, and their weak linkages cause them to be unstable in high temperature and humidity, a common industrial condition (Ma and Scott 2018). Furthermore, their post‐synthesis processing and waste management pose significant environmental challenges. In particular, the presence of metals, especially those from the second (Y‐Cd) and third rows (Hf‐Hg) of the periodic table, is not an issue that can be easily overlooked at large scales. The mentioned limitations can prevent the industrial scalability of MOFs. Although it may be difficult to find a direct substitute with all of the unique properties of MOFs, materials offering similar functionalities with fewer drawbacks are of considerable interest. COFs, which are entirely organic and metal‐free, represent one promising alternative. They combine high surface areas, good structural diversity, and biocompatibility, making them attractive and logical candidates for future research in food packaging. Figure 3a–d presents an integrated analytical framework that goes beyond conventional presentation approaches by simultaneously capturing molecular design principles, advantages, environmental responsiveness, and application‐specific performance metrics of COFs in the context of sustainable food‐packaging innovation.

**FIGURE 3:**
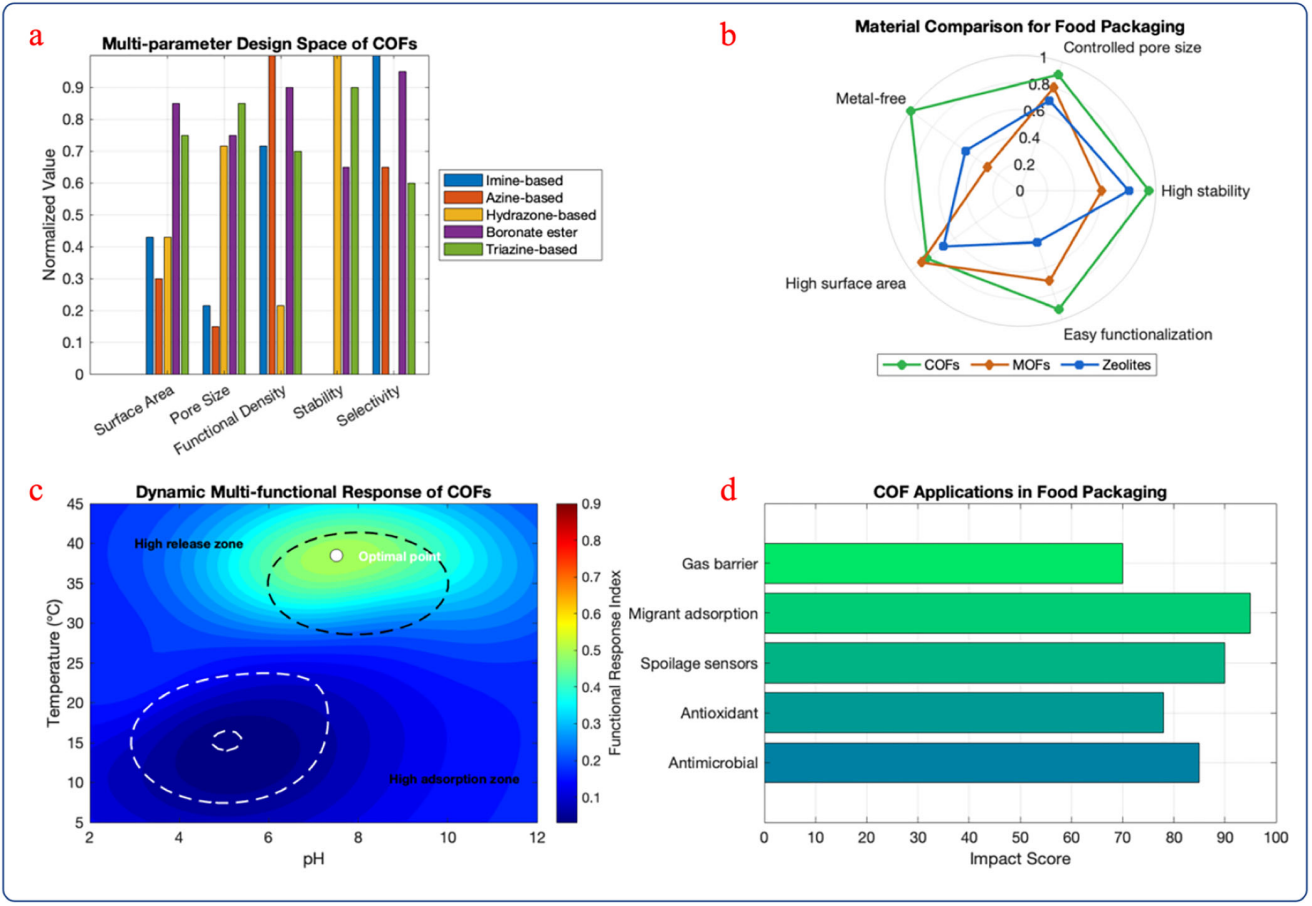
(a) Comparative analysis of key physicochemical parameters across six prominent COF chemistries for food‐packaging applications. The normalized performance metrics reveal distinct property profiles for each COF type: Imine‐based COFs exhibit balanced stability and surface area; azine‐based COFs demonstrate superior functional group density; hydrazone‐based COFs show exceptional stability coefficients; boronate ester COFs offer outstanding selectivity with moderate thermal stability; triazine‐based COFs achieve the highest surface area (up to 2600 m^2^/g) but lower selectivity values; and imide‐based COFs present intermediate characteristics across all parameters. The optimal properties for food‐packaging applications require balanced performance across multiple parameters, with stability and selectivity being particularly critical for ensuring food safety and extended shelf life. (b) Radar plot comparing the relative performance of COFs against other established porous materials (MOFs and zeolites) across five essential characteristics relevant to food packaging. COFs demonstrate superior performance in metal‐free composition (score: 1.0) and controlled pore size (score: 0.90), while maintaining competitive advantages in high stability (score: 0.95) and easy functionalization (score: 0.92). This analysis highlights COFs’ unique position as versatile platforms for next‐generation food packaging, particularly for applications requiring biocompatibility and precise control of molecular interactions at the packaging‐food interface. (c) Dynamic multifunctional response map illustrating the complex interplay between environmental conditions (pH and temperature) and COF performance in food packaging. The contour visualization reveals distinct functional zones: High antimicrobial release (dashed black lines, upper left region) occurs optimally at pH 5–9 and temperatures above 30°C, whereas maximum migrant adsorption capacity (dashed white lines, lower right region) is achieved at pH 4–7 and temperatures below 20°C. The color gradient represents the functional response index (FRI), a composite metric quantifying overall performance. The optimal operational point (white marker) at approximately pH 7 and 25°C indicates conditions where COFs can simultaneously perform multiple functions with balanced efficiency. This visualization demonstrates the adaptability of COF‐based packaging systems to varying storage conditions, offering insight into their performance across typical food supply chains. (d) Impact assessment of COF integration across major food‐packaging applications. Migrant adsorption shows the highest impact score (95), followed by spoilage sensing capability (90), antimicrobial activity (85), antioxidant properties (78), and gas barrier enhancement (70). These scores reflect both the technological readiness level and the potential significance for addressing critical challenges in food preservation and safety. The advanced adsorption capabilities of COFs for harmful migrants from conventional packaging materials represent the most promising near‐term application with immediate implications for food safety and consumer health. COFs: covalent organic frameworks; MOFs: metal–organic frameworks.

### 2.4 Covalent Organic Framework

COFs are crystalline porous polymers constructed entirely from organic building blocks connected through strong covalent bonds, distinguishing them from MOFs, which contain metal nodes, and from conventional polymers, which typically lack permanent porosity. COFs have been well‐documented across various industries, including catalysis, water purification, selective pollutant adsorption, and extraction. Their uniform surfaces, structural stability, precise design flexibility, and reusability are advantages rarely found collectively in other materials. Since their introduction in 2005 (Côté et al. 2005), these porous materials have rapidly evolved, demonstrating a notable edge over MOFs. Although packaging generally does not require harsh conditions, COFs exhibit superior stability and crystallinity compared to MOFs, even under extreme conditions such as strong acids, redox environments, and boiling water (Wei et al. 2025). Studies have shown that integrating COFs, especially those functionalized with groups like amines and acetylenes, with other nanoparticles further enhances their potential for advanced applications (Chen, Wang, et al. 2021). COFs demonstrate superior properties over the commonly used porous materials in the food‐packaging research, including high thermal and chemical stability, precise pore size and shape control, lightweight and metal‐free, high surface area and porosity, easy post‐synthetic modification, and structural adjustability for demanded applications like food packaging (Li, Cai, et al. 2020).

### 2.5 Key Physicochemical Properties of COFs Relevant to Food Packaging

One of the most defining characteristics of COFs is their exceptionally high surface area and precisely tunable porosity, which play a central role in determining their performance in food‐packaging applications. Unlike conventional polymeric materials, which often exhibit random and poorly defined free volume elements, COFs possess highly ordered crystalline architectures with uniform and predictable pore geometries. The specific surface area of COFs typically ranges from 500 to over 3000 m^2^/g, significantly exceeding that of conventional porous polymers and many inorganic materials such as zeolites and silica (El‐Kaderi et al. 2007). This extraordinary surface area dramatically enhances the adsorption capacity of COFs toward gas molecules, volatile organic compounds, and microbial metabolites associated with food spoilage. The pore sizes of COFs can be precisely controlled through rational selection of building blocks and linker geometry, typically ranging from microporous (<2 nm) to mesoporous (2–50 nm) regimes. This precise pore size control enables selective molecular recognition and adsorption of specific molecules such as oxygen, ethylene, ammonia, and biogenic amines, which are critical indicators of food degradation. Furthermore, the ordered pore channels facilitate rapid diffusion and interaction between guest molecules and the internal surface of the framework, enabling efficient sensing and preservation functions. This structural precision also minimizes pore blockage and ensures reproducible functional performance, which is essential for industrial food‐packaging applications where consistency and reliability are critical. Compared to amorphous porous polymers, which often exhibit irregular pore distributions and unpredictable behavior, COFs provide a highly controllable and reproducible platform for functional packaging design. The high internal surface area also enables efficient immobilization and stabilization of active compounds such as antimicrobial agents, antioxidants, and sensing molecules, significantly enhancing their effectiveness while reducing the required loading concentration (Liao and Liu 2021; Heravifard et al. 2022). This property contributes to improved safety and reduced migration risks, as lower concentrations of active agents are required to achieve functional performance. Therefore, the combination of high surface area, uniform porosity, and structural precision represents one of the most important advantages of COFs for next‐generation smart food‐packaging systems.

Another critical property that makes COFs highly suitable for food‐packaging applications is their remarkable chemical and thermal stability. Unlike many conventional porous materials that degrade under humid or chemically reactive environments, COFs are constructed through strong covalent bonds such as imine, hydrazone, boronate ester, triazine, and imide linkages, which provide exceptional structural robustness. This covalent bonding framework enables COFs to maintain their crystallinity, porosity, and functional integrity under a wide range of environmental conditions, including exposure to moisture, temperature fluctuations, and chemical contaminants. In food‐packaging environments, materials are frequently exposed to varying humidity levels, acidic or basic food components, and temperature changes during processing, transportation, and storage. Many traditional porous materials, particularly MOFs, suffer from structural degradation under these conditions due to weaker coordination bonds between metal ions and organic linkers. In contrast, COFs exhibit superior hydrolytic stability and resistance to chemical degradation, making them more reliable for long‐term food contact applications. Certain imide‐linked and triazine‐based COFs have demonstrated stability in boiling water, acidic solutions, and oxidative environments without significant loss of crystallinity or functional performance (Zhang, Lombardo, et al. 2023). Thermal stability is equally important, particularly for packaging processes involving heat sterilization, hot filling, or thermal processing. COFs have demonstrated thermal stability up to temperatures exceeding 400°C, significantly higher than many polymer‐based materials. This high thermal resistance ensures that COF‐based packaging materials maintain their structural integrity and functional properties during manufacturing and sterilization processes. Furthermore, their resistance to photodegradation enhances their durability under light exposure during storage and transportation (Evans et al. 2021; Rahman et al. 2023; Liao and Liu 2021). Chemical stability also plays a critical role in preventing the release of degradation products into food, thereby reducing migration risks and improving consumer safety. The combination of thermal, chemical, and hydrolytic stability ensures that COFs can provide long‐term functional performance in real packaging environments, making them highly promising candidates for industrial implementation.

One of the most unique and valuable properties of COFs is their exceptional structural and chemical tunability, which enables precise customization of their functional performance for specific food‐packaging applications. Unlike traditional porous materials with fixed compositions, COFs can be rationally designed at the molecular level by selecting appropriate organic building blocks and functional groups. This design flexibility allows precise control over pore size, pore shape, surface chemistry, and overall framework functionality. Functional groups, such as amines, hydroxyl groups, carboxyl groups, and aromatic rings, can be incorporated into the framework during synthesis or through post‐synthetic modification, enabling selective interaction with target molecules. This tunability enables COFs to function as highly selective sensors capable of detecting spoilage indicators such as ammonia, hydrogen sulfide, and biogenic amines, which are commonly produced during food decomposition. Additionally, functionalization with antimicrobial agents, such as silver nanoparticles, essential oils, or bioactive molecules, can enhance their ability to inhibit microbial growth and extend food shelf life. Surface modification techniques, such as click chemistry, grafting, and polymer functionalization, further expand the range of possible functionalities. For example, temperature‐responsive polymers grafted onto COF surfaces enable controlled release of preservatives in response to environmental changes, improving preservation efficiency while minimizing chemical exposure (Shen et al. 2023). This ability to precisely engineer functional performance represents a major advantage over conventional packaging materials, which often lack controllable functional properties. Furthermore, the uniform distribution of functional groups within the framework ensures consistent performance across the material, enhancing reliability and reproducibility (Ji et al. 2022; Zhou et al. 2023; Liao and Liu 2021; Qian and Jiang 2024). This tunable nature enables the development of multifunctional packaging systems that combine sensing, preservation, and protective functions within a single material platform, representing a major advancement in food‐packaging technology.

The gas adsorption and tunable pore characteristics of COFs are highly relevant to food packaging because they can contribute to the regulation of gases and volatile compounds associated with food quality. COFs exhibit high surface areas and tailored pore structures that enable interactions with target molecules, supporting multifunctional applications in food packaging. These characteristics can contribute to barrier enhancement, selective molecular capture, and controlled release of active compounds. The specific mechanisms through which these structural and adsorption properties contribute to packaging performance are discussed later (Qian and Jiang 2024; Aksu and Keskin 2025; Liang et al. 2025).

Biocompatibility and safety are among the most critical considerations for food‐packaging materials, and COFs offer significant advantages in this regard due to their metal‐free composition and stable organic structure. Unlike metal‐containing porous materials such as MOFs, which may release toxic metal ions under certain conditions, COFs are composed primarily of light elements such as carbon, hydrogen, nitrogen, oxygen, and boron. This composition significantly reduces the risk of toxic ion migration into food. Additionally, the strong covalent bonding within COFs minimizes structural degradation and prevents the release of potentially harmful components. Numerous cytotoxicity studies have demonstrated low toxicity of COFs at concentrations relevant to food‐packaging applications, with minimal impact on cell viability. Migration behavior is influenced by factors such as particle size, surface chemistry, and interaction with the polymer matrix. Proper integration of COFs within polymer matrices significantly reduces migration risk by immobilizing the particles and limiting direct contact with food. Furthermore, their ability to immobilize active compounds within internal pores reduces the release of preservatives into food, improving safety. Advances in green synthesis and biodegradable COF design further enhance their environmental compatibility (Ghosh and Banerjee 2023; Zhang et al. 2022; Esrafili et al. 2021). However, despite these promising characteristics, comprehensive long‐term safety studies and regulatory evaluations remain necessary before widespread commercial adoption. Understanding migration behavior, biodegradability, and environmental impact will be essential for ensuring safe implementation. The combination of low toxicity, structural stability, and controlled migration behavior makes COFs highly promising materials for safe and sustainable food‐packaging applications.

## 3 COF's Characteristic Features, Classifications, and Synthesis Methods

COFs are formed through covalent bonding between light elements such as hydrogen, boron, carbon, nitrogen, oxygen, and silicon, while adhering to the principles of reticular chemistry (Feng et al. 2012). By strategically tuning their structures and properties, and also by purposefully functionalizing them with various monomers and functional groups, their characteristics, including stability, selectivity, sensitivity, and capacity for controlled adsorption and release, can be tailored. From one perspective, COFs are classified into two main categories: 2D and 3D (Geng et al. 2020) (Figure 4a–k). Each dimension exhibits distinct shapes; 2D COFs are nanosheets or thin films, and 3D COFs have complex structures such as bubbles and blocks. In 2D COFs, covalent bonds are limited to 2D planes with weak interlayer interactions, whereas 3D COFs (which require at least one orthogonal building unit) have a fully interconnected structure through covalent bonds. This leads to the formation of more pores in 3D COFs, and consequently, they are less stable compared to 2D COFs. They also offer a higher specific surface area and lower density than 2D COFs, making them advantageous for practical applications. In general, 2D COFs have only a single, one‐dimensional (1D) integrated channel, whereas 3D COFs have more complex pore structures, including interpenetrating channels and cage structures, which are important in host–guest chemistry (Chen, Huang, et al. 2020; Sasmal et al. 2022). In some cases, 0D and 1D COFs are also classified in this system, which mainly refers to their morphology (Zhao et al. 2024).

**FIGURE 4:**
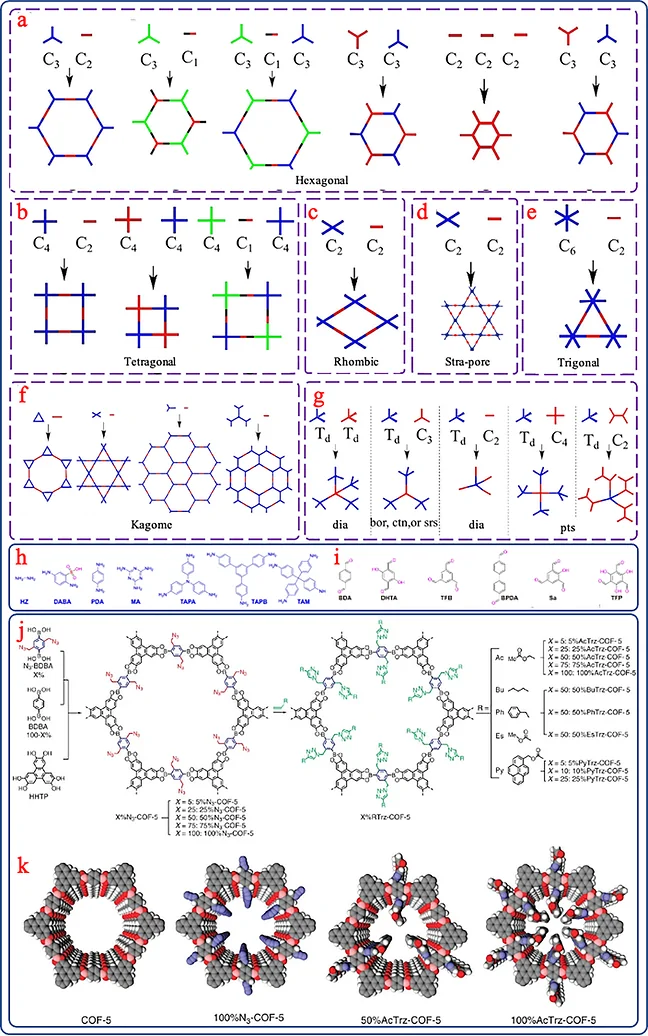
Topological structure of various covalent organic frameworks (COFs): (a) Hexagonal COFs, (b) Tetragonal COFs, (c) Rhombic COFs, and (d) Star‐pore COFs. (e) Trigonal COFs, (f) Kagome COFs, and (g) 3D COFs (Ge et al. 2024). (h) Examples of commonly used amine monomers. (i) Examples of commonly used aldehyde monomers (Asif et al. 2024). (j) The illustration outlines a general approach for surface engineering of COFs by integrating a condensation reaction with click chemistry. In the initial step, COFs containing azide groups on their pore walls are synthesized through the condensation of HHTP with a mixture of azide‐functionalized benzene diboronic acid (N_3_‐BDBA) and regular benzene diboronic acid (BDBA) in a defined molar ratio (*X* = 0%–100%). (k) A schematic of COF‐5 after surface engineering, showing the introduction of organic functional groups onto the pore walls (Nagai et al. 2011). TFP: 2,4,6‐triformylphloroglucinol.

The primary methods for COF synthesis include microwave (MW) synthesis (Chen, Du, et al. 2020; Ding et al. 2020), room‐temperature (RT) synthesis (Guan et al. 2018), aqueous phase‐mediated synthesis (Franco et al. 2020; He et al. 2022), ionic liquids (ILs)‐mediated synthesis (Gao et al. 2019; Zhao et al. 2024), deep eutectic solvents (DESs)‐mediated synthesis (Liu, Qing, et al. 2024), and solvent‐free synthesis (Zhang, Mao, et al. 2023). A crucial initial step in research is the rational selection of a synthesis method based on energy efficiency and environmental considerations. Addressing such constraints significantly affects the scalability and practical applicability of COFs. The most important step in the application of these materials is the rational selection of an appropriate COF, followed by the correct synthesis method. This is because many general properties are shared across different structures, and multiple synthetic routes may exist for a single material. However, selecting the optimal method requires a comprehensive understanding of the synthesis parameters and their implications for the intended application. Xiao et al. (2023) presented a comprehensive approach to synthesis methods and showcased several examples in their study. Table 1 summarizes selected representative COFs along with key features and their corresponding synthesis methods. These examples emphasize key parameters such as high surface area, short synthesis time, water‐based methods, and high synthesis yields, which are bolded in the table. In addition, Wu, Gu, et al. (2025) also focused on RT synthesis techniques for COFs, which help better elucidate their potential for practical use. In addition, effective post‐synthetic modification techniques can be employed to achieve the desired structure and functionalize these frameworks. These methods, aimed at creating well‐structured designs through the introduction of specific functional groups, are referred to as pore engineering or surface engineering. Among the post‐synthesis approaches that have recently attracted attention is click chemistry, which is valued for its high efficiency, process simplicity, and alignment with green chemistry principles (Nagai et al. 2011) (Figure 4). Given the significant potential of COFs in food‐packaging applications, their integration into this field can be both exciting and impactful. At this stage, it is crucial to effectively introduce and highlight the capabilities of this class of materials to researchers in the food‐packaging domain.

**TABLE 1:** Key examples of covalent organic framework (COF) syntheses along with conditions, time, and yields.

Methods	COF type	Reactive monomer	Synthetic conditions	Time	BET surface area (m^2^/g)	Yield (%)	Reference
MWs synthesis	COF‐5	BDBA + HHTP	1,4‐Dioxane/mesitylene, 200 W, 100°C	20 min	2019	68	Campbell et al. (2009)
MWs synthesis	COF‐102	TBPM	1,4‐Dioxane/mesitylene, 200 W, 100°C	20 min	2926	—	Campbell et al. (2009)
	TfpTP‐H	TP‐H + Tp	DCE/1,4‐dioxane/HAc, 250 W, 175°C	10 min	—	83	Vazquez‐Molina et al. (2018)
	TfpTP‐OEt	TP‐OEt + Tp	DCE/mesitylene/HAc, 250 W, 175°C	15 min	—	85	Vazquez‐Molina et al. (2018)
	TfpTP‐OMEG	TP‐OMEG + Tp	DCB/Prop/HAc, 250 W, 175°C	20 min	—	75	Vazquez‐Molina et al. (2018)
Other energy methods	COF‐5	BDBA + HTTP	1,4‐Dioxane/mesitylene, 27.1°C, UV irradiation	24 h	2026.9	75–90	Franco et al. (2020)
RT synthesis	TpBD	Tp + DABP	EtOH, RT	30 min	885	50	Yang et al. (2015)
	N_3_eCOF	HZ + TFPT	DCE/EtOH/HAc, RT	3 days	1722	85	Peng et al. (2016)
	NUS‐14	TFPT + Pa	Mesitylene/EtOH, HAc, RT	3 days	1164	82	Peng et al. (2016)
Aqueous phase‐mediated synthesis	CAF‐1	TMA + CHDA	DMF/H_2_O, 250°C	3 days	256	99.10	Stewart et al. (2017)
	CAF‐2	MTAB + CHDA	DMF/H_2_O, 250°C	3 days	354	92.50	Stewart et al. (2017)
DESs‐mediated synthesis	TpHa‐Gly	Tp + HZ	ChCl/Gly DES, RT	2 h	577	90	Qiu et al. (2020)
Solvent‐free synthesis	TpBD‐Me_2_	Tp + DABP‐Me_2_	170°C, PTSA‐H_2_O	60 s	3109	—	Karak et al. (2017)
	TpAzo	Tp + Azo	170°C, PTSA‐H_2_O	60 s	3038	—	Karak et al. (2017)

Abbreviations: DESs, deep eutectic solvents; MW, microwave; RT, room‐temperature.

## 4 COF Applications in Food Packaging

COFs have the potential to be used in active and smart food packaging for enhancing the shelf life of foods, indicating the degree of freshness or deterioration of foods, and improving the physical and barrier properties of the packaging materials. A Scopus database search reveals that COF applications in food packaging emerged as a research area in 2020, indicating this field's recent development but rapid progress. Several successful applications have been published in various research articles since then. In 2020, a COF (named [HC≡C]0.5‐TPB‐DMTP‐COF) based on imine linkages was synthesized and functionalized with dopamine (as an antioxidant) through a click chemistry method (COFDOPA). The exfoliated form of COFDOPA, designated COFDOPA‐e nanosheets, was successfully integrated into a plasticized poly lactic acid (PLA) matrix alongside acetyl tributyl citrate (ATBC). The inclusion of ATBC increased the flexibility of the films. Both COFDOPA and COFDOPA‐e increased the crystallinity, thermal stability, and mechanical properties of the films. Although non‐exfoliated COFDOPA was released from the packaging material into a fatty food simulant at 1 and 3 wt%, lower concentrations (0.5 and 1 wt%) interacted more efficiently with the polymeric matrix, leading to overall migration levels well below the established legislative limits. The antioxidant activity of dopamine could scavenge the DPPH radical when covalently bonded within COF nanosheets and well‐dispersed in the PLA–ATBC matrix (García‐Arroyo et al. 2020).

The application of Ag NPs has been widely studied in food‐packaging films due to their antibacterial activity. As these NPs are prone to aggregation, which results in lower antibacterial effect, COFs can be used as an efficient vehicle for loading them to enhance their dispersibility and interaction area, as well as fully unlock their functional characteristics. In this regard, Dai et al. synthesized COFs‐Ag NPs by in situ immobilization of Ag NPs within COF‐SCU1 and then incorporated them into chitosan film‐forming dispersion to prepare a film for white crucian carp packaging. They reported a homogenous distribution of uniformly sized Ag NPs within the COFs‐Ag NPs, improved physicochemical properties of the chitosan films (mechanical and UV barrier properties, water solubility, swelling, and water vapor permeability) as a result of incorporating COFs‐Ag NPs, and enhanced quality of the white crucian carp as a result of antibacterial activity (against *Escherichia coli* and *Staphylococcus aureus*) of Ag NPs (Dai et al. 2022). Similarly, Zhou et al. fabricated a chitosan film incorporated with COFs‐Ag NPs to assess its protective and antibacterial effects on green grapes. COF prevented Ag NPs from aggregating and thus enhanced their antibacterial activity. COFs‐Ag NPs composites demonstrated bacteriostatic activity (against *E. coli*, *S. aureus*, and *Bacillus subtilis*) at concentrations between 10 and 20 µg/mL, exhibiting a bacteriostatic rate of 94.01%–98.77%. Furthermore, the addition of COFs‐Ag resulted in improved mechanical and water vapor barrier properties as well as excellent preservation efficacy on green grape samples (Zhou et al. 2025). COFs can also address the challenges associated with the application of essential oils as natural antioxidant and antimicrobial agents in active food packaging, for example, high volatility, low water solubility, and a strong aromatic odor. In research by Shen, thymol essential oil (THM) was loaded within a COF, synthesized using an asymmetric monomer exchange approach. Solution blow spinning was used to prepare the THM‐COF/polycaprolactone (PCL) nanofibrous films. Incorporation of THM‐COF into PCL films led to a film with improved barrier properties, biocompatibility, and antibacterial activity. The THY–COF/PCL films had a temperature‐responsive THY release profile with a sustainable continuous release, making them particularly suitable for practical food preservation applications in warm environments (Shen et al. 2023).

Beyond these early demonstrations, the field has recently advanced toward hybrid COF‐inspired systems that combine low‐cost bio‐derived monomers with rational structural design to achieve multifunctional preservation capabilities. A particularly notable example involves the use of natural lignin as a green monomer to synthesize HCOPs with high crystallinity, permanent porosity, and remarkable thermal stability (Li et al. 2022). When processed into hybrid membranes, these LT‐HCOP materials function as molecular “switches,” finely regulating gas permeability in passive modified atmosphere packaging (MAP) systems. Unlike conventional plastic films, LT‐HCOP membranes integrate multiple protective attributes, antimicrobial, and antioxidant activity, selective CO_2_/O_2_ transport, ultraviolet shielding, and controlled water vapor transmission, thereby establishing an optimal microenvironment that slows microbial proliferation while preventing excessive dehydration. Their effectiveness has been demonstrated through two complementary applications. When employed as a lining material in sealed dishes, LT‐HCOP membranes enabled strawberries to retain nearly 80% edibility after 5 days of storage at RT, compared with rapid fungal decay observed in polyethylene or unprotected controls (Figure 5a–c). Similarly, when used as sealing layers in preservation boxes for waxberries, cherries, and cherry tomatoes, the membranes not only reduced internal condensation but also preserved fruit morphology and extended shelf life by up to 10–12 days. Importantly, these materials retained their functionality over multiple preservation cycles, underscoring their potential for sustainable deployment. Taken together, such developments underscore how COFs and COF‐inspired materials are transitioning from academic concepts to practical food‐packaging solutions. Whether through the stabilization of metal nanoparticles, the controlled release of natural essential oils, or the integration of lignin‐based HCOPs into passive MAP systems, these advanced porous polymers are redefining the design principles of smart packaging. By simultaneously enhancing barrier performance, introducing active antimicrobial and antioxidant functions, and ensuring compliance with safety regulations, COFs and their derivatives are emerging as transformative platforms for tackling the global challenges of food spoilage and waste reduction.

**FIGURE 5:**
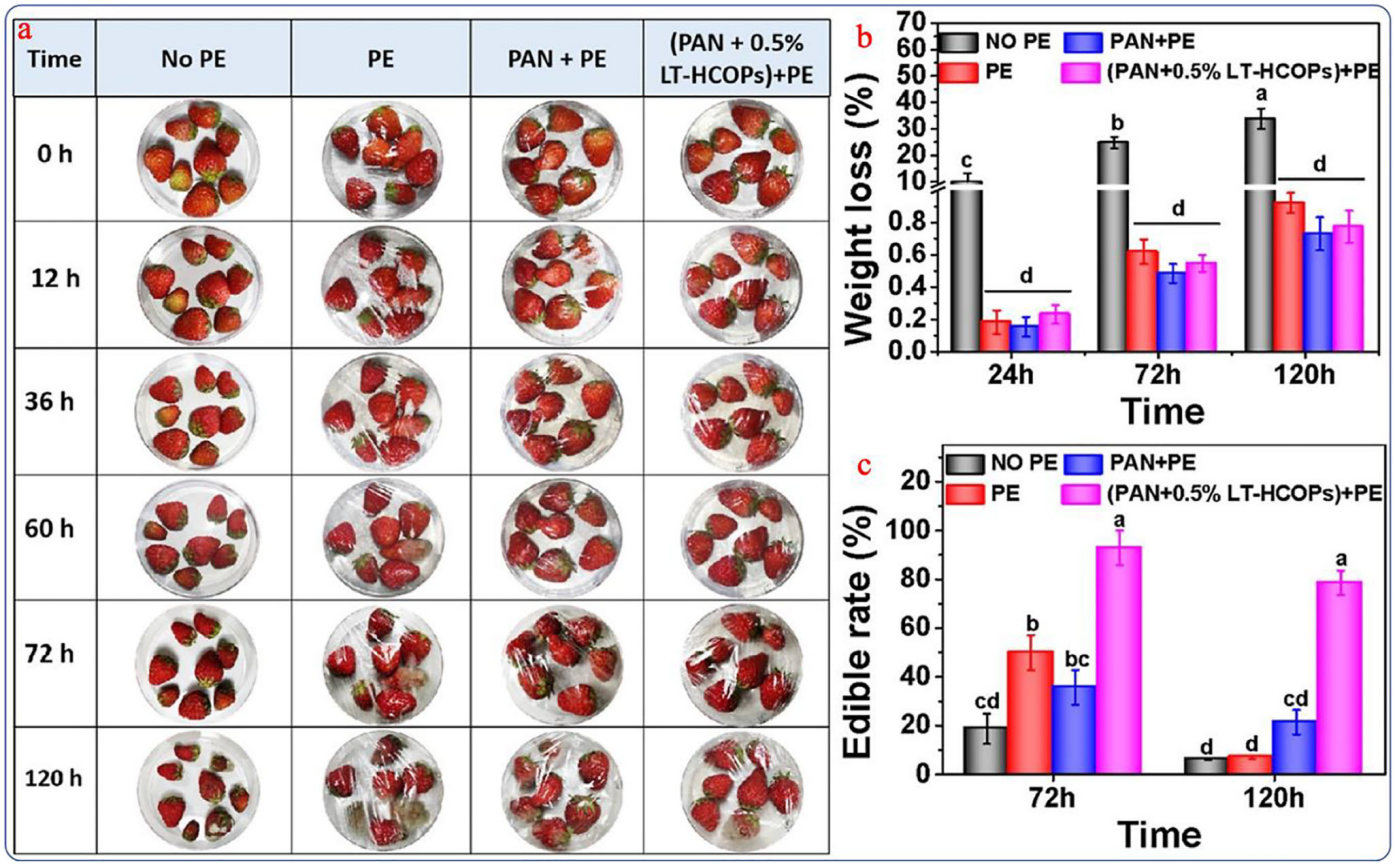
(a) Visual comparison of strawberries stored at ambient conditions over varying storage periods, highlighting the protective role of the hybrid membrane lining. (b) Quantitative assessment of fruit mass reduction during storage, expressed as percentage weight loss over time. (c) Evaluation of strawberry edibility, presented as the proportion of fruits remaining consumable across different storage durations. Distinct alphabetical labels in Panels (b) and (c) denote statistically significant differences between groups at *p* < 0.05, based on Duncan's multiple range test (*n* = 3) (Li et al. 2022). Reprinted with permission from Li et al. (2022), Copyright 2022, American Chemical Society. HCOP: hydrogen‐bonded covalent organic polymer.

In addition to the mentioned benefits of applying COFs in food packaging, COFs can be engineered to provide real‐time monitoring of food freshness. Concerning this, two‐layered silk fibroin (SF)‐COFs (COF_TFPT_ [tetrafluorophenyl terphenyl], COF_BTCA_ [benzene‐1,3,5‐tricarboxylic acid], and COF_DMTA_ [dimethyl terephthalate]) films were developed by growing a layer of COF on the surface of SF film as a smart packaging and labeling solution to tackle multiple challenges encountered throughout the food supply chain in tropical regions (Han et al. 2023). SF‐COF films, specifically SF‐COF_DMTA_ films, showed wrinkled structures that enabled rapid, noticeable color changes in response to putrescine, demonstrating their capability to sense and report spoilage in packaged chicken meat. COF_TFPT_, with its high surface area, was effective in hosting active molecules such as hexanal. This property allowed SF‐COF_TFPT_ films loaded with hexanal to inhibit mold growth in legumes stored under typical tropical climate conditions, characterized by high humidity and temperature that promote rapid microbial growth and spoilage.

In another work, COFs were prepared using 2,4,6‐triformylphloroglucinol (TFP) and *p*‐phenylenediamine (Singh et al. 2024). These COFs were then incorporated into a sodium alginate polymeric material for sensing strips with enhanced colorimetric response and improved mechanical properties and applied in packaged poultry meat, where they exhibited high pH sensitivity and color changes corresponding to the surrounding pH levels. SA‐COF strips exhibit a visible color change in response to biogenic amines released from meat samples, facilitating the detection of food spoilage with the naked eye. The COF's structural properties also allow for the incorporation of citral as a natural antimicrobial agent. When applied as packaging for raw peanuts, its shelf life was extended by inhibiting mold growth under standard temperature and humidity conditions.

Conventional COF powders often exhibit poor dispersibility in aqueous and polar media, restricting their practical deployment in many fields (Li, Jing, et al. 2020; Wang et al. 2019). Addressing this limitation, researchers have recently developed hollow COF nanoparticles (h‐COF NPs) functionalized with water‐soluble polymers, achieving robust dispersibility, thermal responsiveness, and high loading capacities suitable for controlled‐release applications in food preservation (Ma et al. 2022). Specifically, imine‐linked COF hollow nanoparticles were synthesized with an average outer diameter of ∼239 nm, inner cavity diameter of ∼150 nm, and pore width of 1.3 nm. To impart aqueous compatibility and stimuli‐responsiveness, these nanoparticles were grafted with polymers such as polyethylene glycol (PEG) or poly(*N*‐isopropylacrylamide) (PNIPAM) at grafting densities ranging from 0.6 to 2.1 nm^−2^. The resulting h‐COF‐g‐PNIPAM exhibited reversible thermoresponsive behavior: Dispersions remained stable at 25°C but underwent precipitation above 40°C due to the lower critical solution temperature (LCST) of PNIPAM (∼30–35°C). This phase transition was highly reversible, with particle size and polydispersity remaining nearly unchanged over at least 15 heating–cooling cycles, highlighting the robustness and recyclability of the system. Characterization by FE‐SEM, TEM, XRD, FT‐IR, and TGA confirmed the hollow architecture, crystalline framework, and successful polymer grafting, with PNIPAM accounting for ∼4.8 wt% of the nanoparticles. SEM and TEM imaging revealed abundant nanoflakes decorating the particle surfaces, whereas XRD patterns confirmed retention of COF crystallinity post‐grafting. FT‐IR spectra verified the presence of grafted polymer functionalities, and TGA quantified polymer loading consistent with calculated grafting densities. These hollow COF nanoparticles demonstrated exceptional performance as nanocarriers for essential oil preservatives, including hexanal and *trans*‐2‐hexenal, achieving loading capacities up to 1.1 g/g, approximately 60% higher than comparable non‐hollow COF materials. Controlled release studies indicated that encapsulated volatile aldehydes were gradually liberated over extended periods, with only ∼50% hexanal released after 2 days at 25°C, in contrast to near‐complete release from free molecules. When applied to cherry tomatoes, the h‐COF‐g‐PNIPAM loaded with hexanal or *trans*‐2‐hexenal delayed ripening by approximately 4 days compared to controls, demonstrating practical utility in prolonging fruit shelf life (Figure 6a–d). Notably, the nanoparticles could be recollected via thermal‐triggered precipitation, reloaded with essential oils, and reused for multiple preservation cycles without significant loss of efficacy. This work establishes a new class of stimuli‐responsive, hollow COF nanocarriers that combine high dispersibility, recyclability, and tunable release kinetics. By integrating hollow architectures with polymer grafting, these nanomaterials provide a powerful platform for controlled delivery of bioactive agents, highlighting their potential for advancing sustainable and efficient strategies in food preservation and beyond.

**FIGURE 6:**
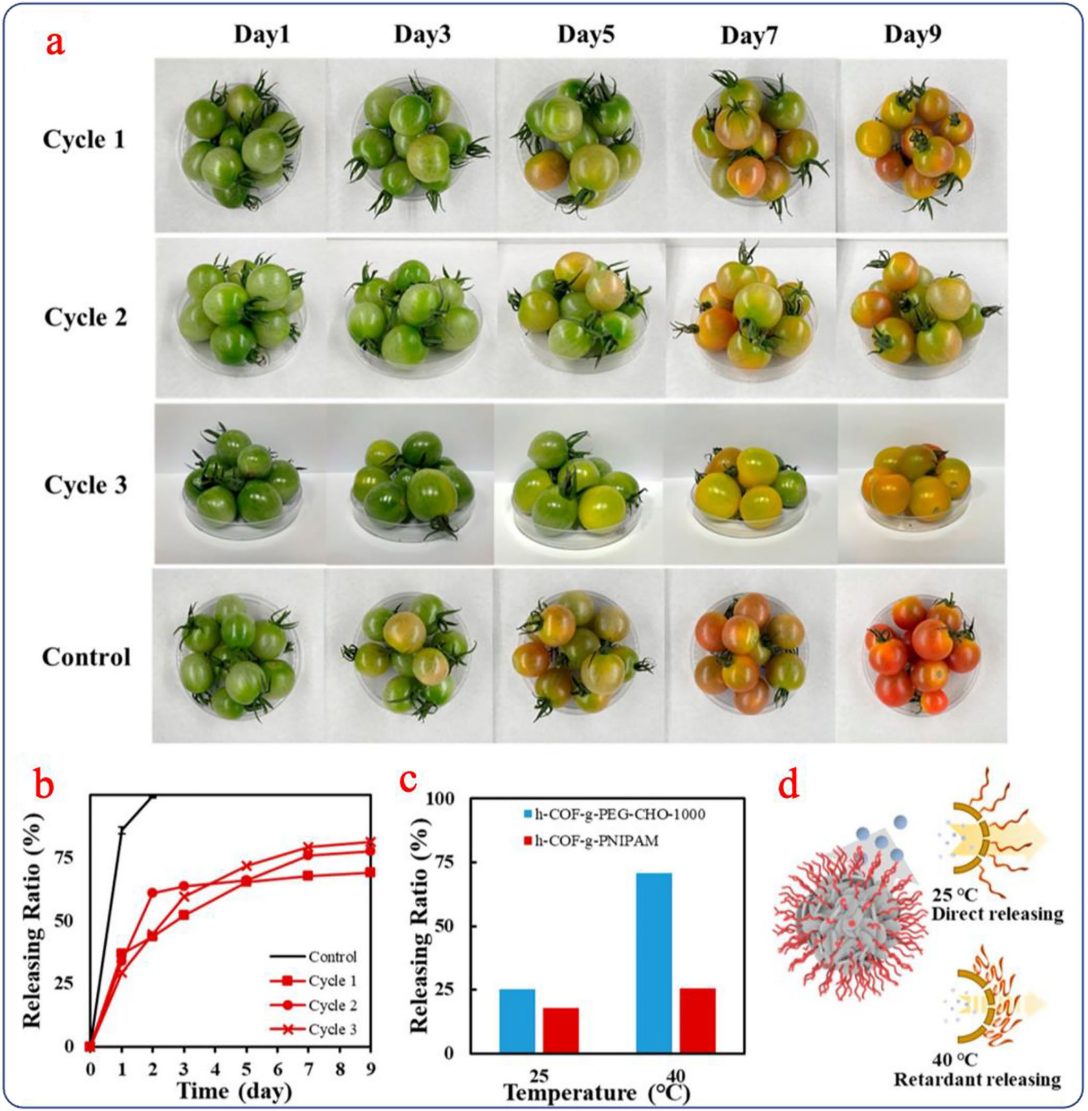
Controlled release and reusability of hexanal‐loaded h‐COF‐g‐PNIPAM nanocarriers. (a) Monitoring the ripening progression of cherry tomatoes treated with hexanal encapsulated within h‐COF‐g‐PNIPAM; after the initial use, the nanocarriers were recovered and replenished with hexanal for two additional testing cycles (Cycles 2 and 3). A reference experiment using an equivalent amount of free hexanal was performed for direct comparison. (b) Time‐dependent release profiles of hexanal from h‐COF‐g‐PNIPAM over three consecutive cycles, with the release from unencapsulated hexanal also plotted for baseline comparison. (c) Comparative evaluation of hexanal release efficiency from h‐COF‐g‐PNIPAM versus h‐COF‐g‐PEG‐CHO‐1000. (d) Conceptual schematic depicting the temperature‐responsive release mechanism of hexanal from h‐COF‐g‐PNIPAM at 25°C and 40°C (Ma et al. 2022). Reprinted with permission from Ma et al. (2022), Copyright 2022, American Chemical Society. COF: covalent organic framework; PEG: polyethylene glycol; PNIPAM: poly(*N*‐isopropylacrylamide).

The innovative incorporation of COFs with abilities of sensing, structural reinforcement, and antimicrobial agent delivery within a biodegradable nanocomposite framework heralds the advent of a new class of active and smart packaging materials. These advanced materials exhibit significant potential in decreasing food waste and increasing food safety standards. A summary of the remarkable results regarding the application of COFs in active and smart food packaging is presented in Table 2.

**TABLE 2:** Covalent organic frameworks (COFs) applied in the food packaging.

COF	Film/Food type	Main COF features	Application/Remarkable results	Reference
[HC≡CH]0.5‐TPB‐DMTP‐COF (adding 27.69 mg of CuI and 100 mg of [HC≡CH]0.5‐TPB‐DMTP‐COF p in a mixture of THF/H_2_O (13 mL; 3/1) and purging with Argon and then adding *N,N*‐dii‐sopropylethylamine (DIPEA), (87 µL, 0.50 mmol))	Poly lactic acid	− COFDOPA was stable up to 300°C − Incorporation of dopamine into the COF decreased BET surface area and pore volume − COFDOPA had a monomodal size distribution in 90 min of sonication (6 cycles of 15 min), with an average value of 255 nm, ranging from 190 to 300 nm − Transparent COFDOPA‐e nanosheets with lateral dimensions around 100 nm as observed by TEM	Designed for non‐migrating active packaging applications/ − Enhancing crystallinity, thermal stability, and mechanical properties of the films − Overall migration levels below the established legislative limits at 0.5% and 1% concentrations	García‐Arroyo et al. (2020)
COF‐SCU1 (dissolving trimesoyl chloride (4.24 g,16 mmol) and *p*‐phenylenediamine (1.28 g, 12 mmol) 120 and 60 mL ethyl acetate, and then adding the *p*‐phenylenediamine into trimesoyl chloride solution)	Chitosan/white crucian carp	− Spherical particles with a certain degree of adhesion − Ag were uniformly distributed in the COFs‐Ag NPs composites − Amorphous structure of COF‐SCU1 − Surface charge of COF‐SCU1 was −60.5 mV and that of COFs‐Ag NPs was −46.5 mV	Carrier of Ag nanoparticles/ − Increasing Ag nanoparticles dispersibility, interaction area, and functional characteristics (antibacterial effect) − Improving mechanical and UV barrier properties, water solubility, swelling, and water vapor permeability of chitosan films − Higher quality of the food product (white crucian carp)	Dai et al. (2022)
TPB and DVA (dissolving 1.4 mg of TPB and 1.1 mg of DVA a 5 mL acetonitrile)	Chitosan/Green grape	− Uniformly dispersed Ag NPs on the surface of the COFs − Ag NPs (3–10 nm) black granules attached to the surface of the COFs − COF particle size ∼300 nm	Carrier of Ag nanoparticles/ − Increasing Ag nanoparticles dispersibility, interaction area, and antibacterial effect − Increasing mechanical and barrier properties and decreasing hydrophilicity of the films − Excellent preservation effect on green grape	Zhou et al. (2025)
TDP‐COF (adding 9.75 mg of BTCA,182.1 µL of benzaldehyde, 163.2 µL of aniline, and 0.2 mL of 1,4‐dioxane/mesitylene (2/1, v/v) containing 1.5 mg of Sc (OTf)3 used as a catalyst into 30.0 mL of 1,4‐dioxane/mesitylene)	Polycaprolactone (PLC) nanofibrous film	− THY loading capacity of 30.35% for THY@COF − Porous crystal structure with an irregular surface. For COFs and THY@COFs − Size of 107.3 and 110.0 nm, for COFs and THY@COFs, respectively. − Similar FT‐IR spectra for THY@COF and COF − Highly crystalline structure of THY@COF − THY@COF gradually started to degrade after 200°C, leaving 56.74% and 56.08% residual weight by the end of heating good thermal stability of the COF and THY@COF − Surface area of 366.716 cm^2^/g	Temperature‐responsive release of thymol in active packaging/ − COF loading capacity 30.35% for thymol essential oil − Increasing yellowness and water vapor barrier property, decreasing mechanical properties, appropriate hydrophobicity of the PLC films − Temperature‐responsive sustained release of thymol − More release at 37°C compared to 4°C − Increasing antibacterial effect with increasing THY–COF concentration − Nontoxicity of the COF	Shen et al. (2023)
COF_TFPT_, COF_BTCA_, and COF_DMTA_ (F‐COF film was fabricated by in situ growth of COF on SF film: Submerging 15 mL of mesitylene/dioxane (v/v, 1/1) mixed solvent with 10 mM BTCA/TFPT/DMTA and 0.6 mM Sc(OTf)_3_ and then adding 15 mL of mesitylene/dioxane (v/v, 1/1) mixed solvent with 10 mM BTCA/TFPT/DMTA, 120 mM aniline, and benzaldehyde)	Silk fibroin (SF)/Chicken meat and Soybean	− High crystallinity and surface area − Stable at high temperature and exhibit high resistance to water	Sensing food spoilage in smart packaging; antimicrobial active packaging/ − Good mechanical durability, resistant to long‐time storage, adhesive tape sticking, bending, and scratching and good transparency − Appropriate soil biodegradability − Immediate colorimetric response to putrescine for poultry − Decreased biotic spoilage in high temperature and humidity condition for soybean by hosting hexanal	Han et al. (2023)
2,4,6‐Triformylphloroglucinol (TFP) and *p*‐phenylenediamine (0.1 mmol of 2,4,6‐trihydroxybenzene‐1,3,5‐tricarbaldehyde and 0.3 mmol of 1,4‐phenyl‐enediamine)	Sodium alginate/poultry meat and Peanut	− Highly amorphous 2D structure and π−π stacking between the COF layers − Formation of an imine bond between TFP and *p*‐phenylenediamine − Porous flower‐like structures with pore sizes varying from 2 to 10 nm − Total pore volume of 1170 cm^3^/g, adsorption cross‐section area 0.163 nm^2^, density 0.809 g/cm^3^, and mean pore diameter 2.9569 nm	Sensing food spoilage in smart packaging; antimicrobial active packaging/ − Improved mechanical properties and appropriate biodegradability − Monitor poultry meat spoilage in different − pHs by colorimetric responses − increases peanut shelf − life by preventing mold formation in typical − conditions of temperature and humidity through inclusion of citral in the COF	Singh et al. (2024)

### 4.1 Barrier Enhancement Mechanism of COFs in Food‐Packaging Systems

The incorporation of COFs into polymer‐based food‐packaging materials can enhance their barrier performance through several complementary mechanisms, including increased diffusion tortuosity, adsorption‐mediated diffusion retardation, and polymer–filler interfacial interactions. In conventional polymer films, gas transport typically occurs through a solution–diffusion mechanism, where gas molecules dissolve into the polymer matrix and diffuse along relatively direct pathways driven by concentration gradients (Kausar 2025; Abedi‐Firoozjah et al. 2025; El Guerraf et al. 2024). The incorporation of COF particles can disrupt these diffusion pathways, requiring gas molecules to follow longer and more complex routes around the framework domains. This increase in diffusion path length may reduce effective diffusivity and consequently contribute to lower gas permeability. The extent of this effect depends on factors such as COF morphology, aspect ratio, loading concentration, and dispersion within the polymer matrix.

In addition to modifying diffusion pathways, the highly ordered nanoporous structure of COFs provides adsorption sites that can interact with permeating molecules such as oxygen, carbon dioxide, ethylene, and water vapor. Depending on the pore environment and functional groups, these interactions may involve physical adsorption or stronger specific interactions. Adsorption within COF pores can temporarily retain permeating molecules and may reduce their effective mobility, thereby contributing to delayed transport through the packaging material. The selective adsorption capability of COFs is particularly relevant to ethylene management because ethylene is associated with ripening and senescence in fruits and vegetables. COFs can selectively adsorb ethylene through their tailored pore sizes and chemical functionalities, thereby potentially reducing its concentration within the packaging environment and contributing to delayed ripening processes (Li et al. 2026; Guo, Guo, et al. 2025; Cheng et al. 2020; Hossain et al. 2024).

The polymer–COF interface can also contribute to barrier enhancement. Strong interfacial adhesion between COF particles and polymer chains may reduce the formation of interfacial voids that could otherwise provide preferential pathways for gas transport (Liu et al. 2015). The surface chemistry of COFs can be tailored to improve compatibility with specific polymer matrices, potentially promoting more homogeneous dispersion and reducing particle aggregation. In addition, interactions between COFs and neighboring polymer chains may restrict local polymer‐chain mobility and reduce the accessible free volume within the matrix. Because gas diffusion is influenced by polymer free volume, these interfacial and confinement effects may further contribute to reduced permeability.

Moisture transport is another important aspect of barrier performance in food packaging. Water vapor transport through polymeric materials generally involves diffusion and sorption processes. COFs can modify water vapor permeability depending on their hydrophilicity, surface chemistry, and functionalization (Li, Cai, et al. 2020). Hydrophobic COFs may reduce water uptake and permeability by limiting water sorption, whereas hydrophilic COFs may interact more strongly with water molecules and potentially contribute to humidity regulation through adsorption and release. Such moisture‐related effects may be relevant to maintaining suitable storage conditions and limiting quality deterioration associated with excessive moisture or dehydration.

The effectiveness of COFs as barrier enhancers is strongly influenced by structural and compositional factors, including particle size, aspect ratio, pore structure, loading concentration, and dispersion quality. Smaller particle sizes and appropriate aspect ratios may provide more effective modification of diffusion pathways when COFs are uniformly distributed within the polymer matrix. However, excessive loading may promote particle aggregation, which can reduce barrier efficiency and adversely affect mechanical properties. Therefore, optimizing COF loading concentration and maintaining homogeneous dispersion are important for achieving effective barrier performance. Quantitative studies have demonstrated significant reductions in gas permeability following the incorporation of COFs into polymer matrices. For example, oxygen permeability reductions of 30%–70% have been reported depending on COF structure and loading concentration (Cao et al. 2020; Liu et al. 2025; Singh et al. 2024; García‐Arroyo et al. 2020).

Beyond passive barrier enhancement, COFs can also function as active packaging components by enabling the controlled release of antimicrobial agents or antioxidants. Their porous structures may serve as carriers for active compounds, whereas interactions between the COF framework and incorporated agents can influence their retention and release. These active functions may complement passive barrier protection by contributing to the inhibition of microbial growth and oxidative deterioration. The barrier performance of COF‐based packaging can result from the combined effects of diffusion‐pathway modification, molecular adsorption, polymer–COF interfacial interactions, free‐volume regulation, and selective molecular capture, with their relative contributions depending on the COF structure and its integration within the polymer matrix (Shen et al. 2023).

### 4.2 Effect of COF on the Mechanical Properties of Food‐Packaging Materials

The incorporation of COFs can substantially modify the mechanical properties of food‐packaging films, although the direction and magnitude of this effect depend strongly on COF content, dispersion, morphology, and COF–polymer interactions. At appropriate loading levels, well‐dispersed COFs can act as reinforcing nanofillers by increasing the compactness and homogeneity of the polymer network and promoting strong interactions with polymer chains. For example, in HPMC/TZP‐COF films, hydrogen bonding and van der Waals interactions between the COF and HPMC matrix enhanced intermolecular interactions, restricted polymer‐chain slippage, and resulted in simultaneous increases in tensile strength and elongation at break at low COF concentrations (García‐Arroyo et al. 2020). Similar reinforcement has been reported for chitosan films containing COF‐Ag NPs, where the nanocomposite uniformly occupied free spaces within the polymer network, whereas cross‐linking and molecular entanglement increased film rigidity and tensile strength (Dai et al. 2022). In contrast, increasing the COF content beyond an optimum level can promote particle aggregation and generate stress‐concentration sites, leading to premature chain fracture and deterioration of mechanical performance, as observed at higher TZP‐COF loading in HPMC films (García‐Arroyo et al. 2020). The effect can also be matrix‐dependent; incorporation of COF or thymol‐loaded COF into PCL nanofibers decreased tensile strength and elastic modulus, which was attributed to disruption of nanofiber continuity, although elongation at break increased markedly, indicating greater ductility (Shen et al. 2023). Similarly, COF‐Ag NPs incorporation into carboxymethyl chitosan increased tensile strength while reducing elongation at break, demonstrating increased mechanical resistance at the expense of flexibility (Qiu et al. 2025). Changes in crystallinity may provide an additional reinforcement mechanism, as reported for starch films containing COF‐based active components, where increased crystallinity was associated with higher tensile strength and Young's modulus (Cao et al. 2025). These studies indicate that COFs can function either as reinforcing or defect‐generating fillers depending on their concentration and compatibility with the polymer matrix, and therefore optimization of COF loading, dispersion, interfacial interactions, and morphology is essential for achieving the desired balance between strength, stiffness, and flexibility in food‐packaging films.

## 5 Challenges and Opportunities

### 5.1 Safety Important Factors

Assessing the safety of new materials, such as COFs, is crucial, with a particular focus on their biocompatibility and potential toxicity to determine whether a material can perform its intended function without causing harm to human health, including immune response and inflammation, as well as their potential effects on the environment. A comprehensive understanding of these aspects will contribute to the responsible development and implementation of COFs in various applications, ensuring minimal risk to both human health and the ecosystem (Alizadeh Sani et al. 2024). The current study underscores the significant potential of COFs for utilization in food‐packaging materials. However, maintaining product quality and consumer safety necessitates the absence of any interaction between packaging material and food matrix throughout a product's shelf life (Alizadeh Sani et al. 2024). Regulatory authorities usually impose restrictions on packaging materials that come into direct contact with food (food contact materials [FCM]) that must adhere to stringent nontoxicity requirements to ensure the safety of consumers by minimizing potential health risks associated with the migration of harmful substances from packaging materials into food. Therefore, a thorough evaluation of the safety aspects of COFs is essential before their extensive adoption in food packaging (Han et al. 2023). COFs are composed of hydrogen, carbon, and nitrogen, with their structure comprising inert organic groups linked together by Schiff base bonds. These Schiff base connections are commonly found in plants and natural polymers, highlighting the potential biocompatibility and environmental friendliness of COFs (Han et al. 2023). However, COFs can be synthesized in a complex form with metals through different methods such as metal doping, integration of metal‐anchored linkers, and metal coordination to have unique functionality. The potential risks stemming from the release of metal ions or organic ligands from COFs into the environment or food products should be diligently assessed and accounted for. By addressing these concerns, the promising potential of COFs in food‐packaging applications can be responsibly and sustainably realized (Alizadeh Sani et al. 2024). The safety and biocompatibility of COFs are influenced by several factors, including their composition, structure, size, synthesis method, the solvents used during synthesis, and how they interact with biological systems. Furthermore, the administration method and the manner in which COFs engage with biological environments play a critical role in determining their overall safety and compatibility. Careful evaluation and tailoring of these factors will contribute to the development of safe, biocompatible COFs for food‐packaging applications (Alizadeh Sani et al. 2024).

The nanoscale particle size of COFs enables them to be invasive in nature. The pores within these frameworks can be occupied by solvents such as dimethylformamide (DMF), water, dimethyl sulfoxide (DMSO), acetone, and diethylformamide (DEF). Interestingly, even after activation processes like vacuum treatment, heat treatment, or solvent exchange, which are designed to remove guest molecules from the framework pores, residual solvents may still remain within the COF structure. These remnant solvents can potentially influence the behavior, properties, and safety of COFs in food packaging. Careful consideration and evaluation are needed during material design, synthesis, and post‐synthesis treatments (Sharanyakanth and Radhakrishnan 2020a). In addition, COFs synthesized through various routes typically pose challenges for recyclability due to safety concerns associated with their components, such as metal ions and organic linkers. Consequently, the nonrecyclable nature of COFs must be taken into account when assessing their suitability and sustainability, necessitating a careful evaluation of the potential implications for both human health and the environment. Some COFs (TpBD‐Me_2_) have demonstrated stability in in certain media without compromising their crystallinity or morphology. This stability allows for regeneration using organic solvents, enabling the reuse of the material in consecutive adsorption cycles without compromising its efficiency. This finding highlights the potential of COFs for various applications, where material stability, reusability, and low toxicity are essential (Romero et al. 2019; Salonen et al. 2017).

Ongoing research efforts aim to develop safer, more biodegradable, and recyclable COF materials to overcome these challenges and unlock their full potential in biomedical and other applications (Rajkumar et al. 2019). One promising approach is to employ edible ingredients such as cyclodextrin (CD), tannic acid, and flavonoids in the synthesis of COFs, thereby reducing potential hazards. Additionally, it is vital to adopt RT fabrication methods and utilize precursor materials with benign chemical characteristics. This can be achieved by implementing green synthesis strategies for COFs. For synthesis of COF‐metal complexes, incorporating biocompatible metal ions such as calcium (Ca), potassium (K), and titanium (Ti) is suggested. Moreover, employing biologically acceptable linkers, like peptides, carbohydrates, amino acids, and CD derivatives, can help minimize risks associated with COFs, ultimately paving the way for safer, more sustainable materials (Rajkumar et al. 2019).

Current research on the toxicity of COFs remains limited, with most studies focused on in vitro human cytotoxicity assays that primarily assess cell viability. In in vitro cytotoxicity studies, various macrophages or cell lines are cultured in the presence of a COF suspension for a designated duration. Following incubation, calorimetric detection is employed to analyze the culture media for compounds released due to cell lysis. These compounds interact with specific reagents used in the cytotoxicity test, allowing for the quantification of cytotoxic effects. MTT (3‐(4,5‐dimethylthiazol‐2‐yl)‐2,5‐diphenyltetrazolium bromide), CCK‐8 (Cell Counting Kit‐8), and Alamar Blue can be used for in situ cytotoxicity testing of COFs (Sharanyakanth and Radhakrishnan 2020b). Toxicity assessment at the systematic level using model organisms can also be used for monitoring the dose−response toxicity of COFs (Han et al. 2023). The majority of toxicity assays of COFs have been conducted within the scope of drug delivery, imaging, and photothermal therapy applications (Rezvani Alanagh et al. 2020; Ding et al. 2021). Although some COFs have demonstrated low cytotoxicity against normal or tumor cells in studies related to chemotherapeutical treatment (Sárria et al. 2021), most investigations have shown almost harmless effects at concentrations up to 100 µg/mL (Mi et al. 2019; Vyas et al. 2016). Additionally, an in vivo study using COFs as therapeutic agents revealed that injecting 5 mg/kg per mouse every 3 days or 10 mg/kg did not result in significant toxicity, indicating their potential for safe usage (Zhang et al. 2019). The number of reports is on the cytotoxicity of COFs in food packaging that is limited. Shen et al. assessed the cytotoxicity effect of PCL nanofibrous films containing COF as a carrier for THM by MTT assay and human foreskin fibroblast (L929) cells. Different concentrations of sterilized samples (PCL, COF–PCL, and thymol–COF–PCL nanofibrous films) were added to the cultured cell lines in a 96‐well plate and incubated at 37°C. After adding DMSO to the wells, absorbance was measured at 570 nm. On the basis of the results, negligible cytotoxicity was observed for the samples, including the COF at concentrations in the range of 25–400 µg/mL (Shen et al. 2023). Cytotoxicity assay of Ag NPs, COFs, and their combination (with the potential application in food packaging) on LO_2_ cells indicated that Ag NPs had the highest toxicity against the cells, whereas COFs showed lower toxicity. An increase in the concentration led to a decrease in the cell viability, so that at 40 µg/mL of Ag NPs and COF, cell viability reached below 20% and 58.5%, respectively. It was interesting to observe that the combination of Ag NPs and COF decreased cytotoxicity considerably. Moreover, increasing the concentration of Ag NPs–COF had a slight effect on cell viability, so that at 5–40 µg/mL concentrations, the cell viability was around 73% (Zhou et al. 2025).

Although COFs have the ability to reduce toxicity effects against cells in a composite form compared to the individuals, it is recommended to assess the toxicity impact of each component of the composite due to the possible decomposition and collapse of the composite structure in the food packaging over time, leading to more migration into foods and ecosystems. To investigate the potential risks associated with the release of COFs from packaging materials into the environment, especially aquatic ecosystems, Han et al. conducted cytotoxicity assay of COF against *Artemia salina* (a species of brine shrimp). For this goal, they incubated *A. salina* active nauplii with three types of COFs (COF_BTCA_, COF_TFPT_, and COF_DMTA_) at 100–2000 µg/mL concentrations in the presence of seawater. Counting the live shrimps after 24 h indicated an average mortality rate below 30% for all samples and LC_50_ considerably higher than 2000 µg/mL, which implied on low acute toxicity toward A. salina under the tested conditions. However, the specific cause of the limited mortality was not established, and these findings should therefore be interpreted as preliminary evidence of low acute toxicity rather than definitive evidence of environmental safety. Although these studies provide valuable insights into the potential risks and biocompatibility of COFs, there is a need for further research to fully understand their long‐term effects, in vivo behaviors, and potential impacts on both human health and the environment when using in food packaging. As the development and application of COFs continue to expand, addressing these knowledge gaps will be essential in ensuring their safe and responsible implementation across various fields (Sárria et al. 2021).

Building upon the safety considerations outlined above, it is also critical to evaluate the interaction of COFs with chemical contaminants that may be present in food. Functionalized COFs can serve not only as passive barriers but also as active adsorbents for hazardous substances such as mycotoxins and persistent organic pollutants. For instance, recent studies have demonstrated the use of fluorinated COFs as highly efficient sorbents for per‐ and polyfluoroalkyl substances (PFASs) and aflatoxins in food matrices (Fang et al. 2025). By leveraging the tunable pore structure, functional group density, and electrostatic properties of COFs, these frameworks can selectively capture trace contaminants at sub‐nanogram levels, offering a dual function of food protection and contaminant monitoring. Optimization of critical parameters, such as eluent type, solvent volume, sample loading rate, and solution volume, has been shown to substantially enhance adsorption efficiency while maintaining high analytical sensitivity and recovery rates. For example, TF‐COFs functionalized with quaternary ammonium and fluorinated groups exhibited adsorption capacities up to 44 mg/g for aflatoxins, whereas careful selection of acetonitrile as the eluent enabled minimal dilution and maximal desorption (Figure 7a–l). Similarly, sample loading rates and volumes were fine‐tuned to balance analysis speed with adsorption efficiency, ensuring reproducible and practical applications in food safety monitoring. These findings underscore that, beyond structural biocompatibility and minimal cytotoxicity, COFs can be strategically designed as active food‐packaging materials that not only maintain product quality but also mitigate chemical risks. Integrating such adsorption functionalities with existing safety frameworks reinforces the responsible development of COF‐based packaging systems, highlighting their potential as multifunctional, sustainable materials for future food protection.

**FIGURE 7:**
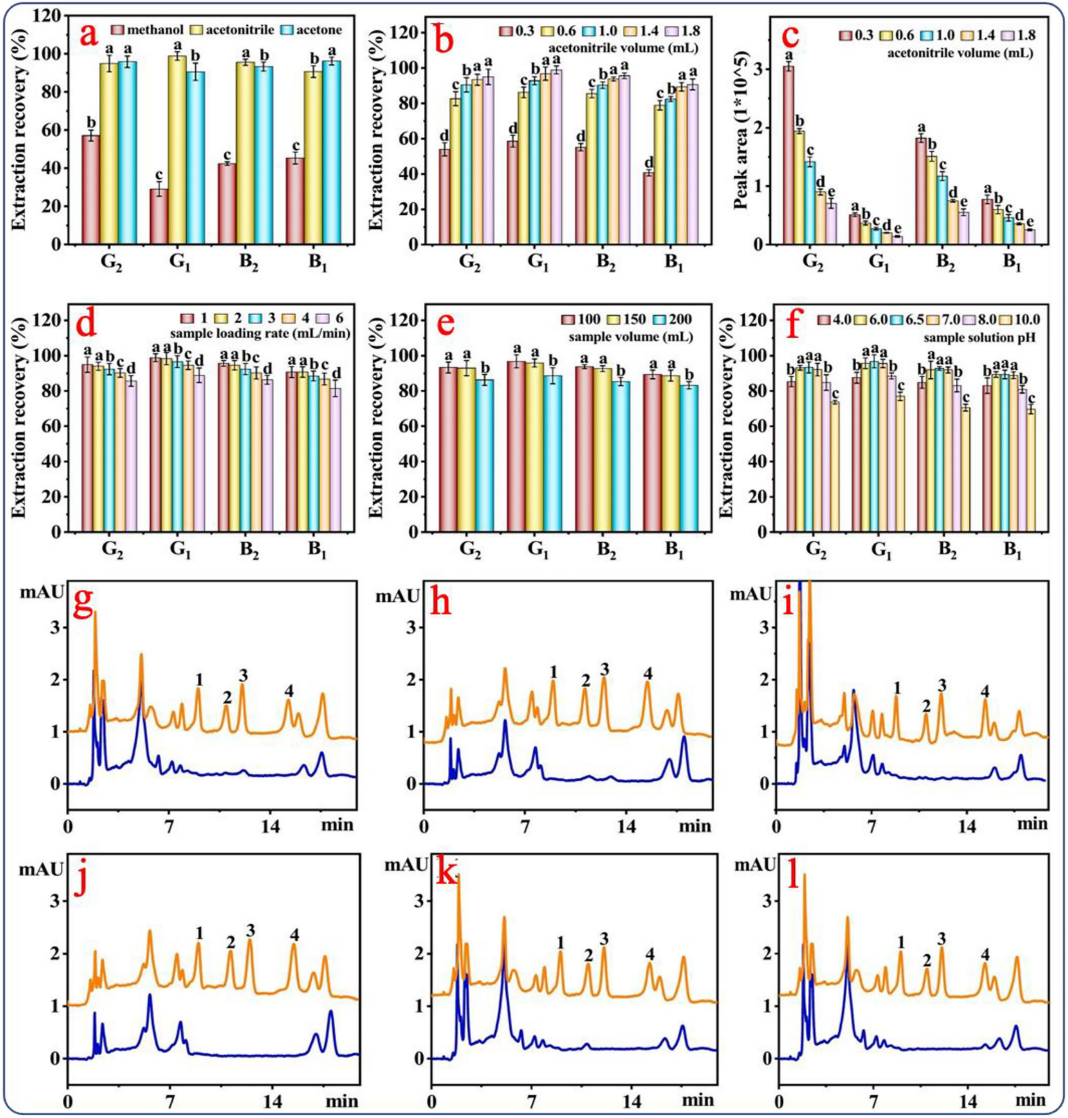
Evaluation and optimization of solid‐phase extraction (SPE) parameters (a–f) and representative chromatograms of peanut samples (g–l). Panel (a) illustrates the effect of different eluent types, (b and c) the influence of acetonitrile volume, (d) sample loading rate, (e) total sample solution volume, and (f) sample pH on extraction efficiency. Panels (g–l) present chromatograms of six individual peanut samples, with the lower trace representing unspiked blanks and the upper trace corresponding to spiked samples at concentrations of 0.20, 4.00, 0.10, and 2.00 ng/g for G2, G1, B2, and B1, respectively. Peaks labeled 1–4 correspond to analytes G2, G1, B2, and B1. In Panels (a–f), different lowercase letters indicate statistically significant differences in extraction performance (*p* < 0.05), whereas identical letters denote no significant difference (*p* > 0.05) among the tested conditions (Fang et al. 2025). Reprinted with permission from Fang et al. (2025), Copyright 2025, American Chemical Society.

### 5.2 Sustainability, Biodegradability, and End‐of‐Life Management of COF‐Based Packaging

Sustainability has three different aspects: human well‐being sustainability, economic sustainability, and environmental sustainability. The aim of these different aspect of sustainability is to enhance human welfare, ensure fair burden‐sharing, and maintain social equity while preserving the resilience of ecosystems. From an ecological viewpoint, it is essential to focus on maintaining a healthy environment and ecosystem, which can also be applied to packaging materials (Boz et al. 2020). Packaging materials are considered sustainable if they reduce the use of virgin resources, are recyclable, or can be reused from readily available materials. Material sustainability is influenced by various factors, including economic and environmental aspects like cost, impact, functionality, aesthetics, production, processing, and end‐of‐life effects at both local and global scales. Some authors argue that achieving absolute sustainability is a significant challenge and may be nearly impossible (Boz et al. 2020).

Biodegradability is an important property that contributes to the environmental aspect of sustainability. The toxicity of nanomaterials is affected by their biodegradability, metabolization, and clearance after their consumption. The rate at which nanomaterials are eliminated depends on the specific degradation mechanism involved. These findings underscore the importance of considering biodegradability when designing nanoCOFs for food‐packaging materials, as it plays a significant role in determining the overall safety and efficacy of these materials (Singh et al. 2023).

Most research on degradable COFs has focused on biomedical applications, especially drug delivery and wound treatment. This body of work shows that degradability can be built into COF frameworks by incorporating stimulus‐responsive linkages. For instance, a ferrocene‐containing COF built on dynamic boroxine bonds broke down when exposed to acidic or H_2_O_2_‐rich environments (Sun et al. 2023). In a comparable case, a porphyrin‐based COF linked through disulfide bonds disassembled in response to glutathione‐triggered bond cleavage (Li, Wan, et al. 2023), and an azo‐linked COF was engineered to decompose via an Fe/H_2_O_2_‐driven oxidative pathway (Li, Wang, et al. 2023). Additional porphyrin‐based COFs featuring ester or hypoxia‐sensitive azo linkages have likewise been developed as biodegradable platforms for wound healing, with their breakdown and clearance assessed through in vitro and/or in vivo experiments (Sun et al. 2021; Liu, Li, et al. 2024). Taken together, this evidence shows that degradability in COFs can be achieved by embedding chemically labile or stimulus‐responsive bonds into the framework; however, these findings largely reflect degradation triggered by biological conditions rather than genuine environmental biodegradation under soil, composting, freshwater, or marine settings.

By comparison, research directly addressing COF biodegradability within food‐packaging contexts is still quite scarce. Several recent efforts have embedded COFs into biodegradable substrates—including SF, PLA, sodium alginate, and carboxymethyl chitosan—to produce biodegradable composite packaging (Han et al. 2023; García‐Arroyo et al. 2020; Singh et al. 2024; Qiu et al. 2025). Yet in most of these cases, the biodegradability reported pertains to the polymer matrix or the composite as a whole, rather than being experimentally confirmed for the COF framework itself. Going forward, studies should independently assess the degradation behavior of the COF component and the polymer component and clarify how the chemistry of COF linkages, the framework architecture, and any functionalization shape their environmental fate under realistic end‐of‐life scenarios.

Managing end‐of‐life disposal and achieving circularity remain significant challenges for COF‐based packaging materials. Although dedicated recycling infrastructure for COF‐containing food‐packaging composites remains limited, emerging research points to promising approaches for integrating COFs into circular material cycles. Notably, chemical recycling of imine‐linked COFs has been achieved through reversible depolymerization followed by reassembly, recovering the original framework at yields of up to 92% while preserving its crystallinity and pore structure (Jing et al. 2026). Life cycle assessments (LCA) have also shown that precursor synthesis and solvent use can substantially contribute to the environmental footprint of COF manufacturing, underscoring the need for greener synthetic and processing strategies (Espada et al. 2023). However, recycling is inherently more challenging for food‐packaging applications because COFs are typically embedded within polymer matrices, which complicates their selective recovery. Future research should therefore address the recyclability of complete COF–polymer composites, together with strategies for material recovery, chemical recycling, solvent reclamation, and compatibility with existing packaging‐waste infrastructure. In this context, LCA provides an important framework for evaluating environmental sustainability across the life cycle of COF‐based materials, including production, use, maintenance, and disposal. However, quantitative LCA studies on selected COF systems have highlighted the environmental impacts associated with their synthesis and application, including global warming potential, stratospheric ozone depletion, and human toxicity potential (Ge et al. 2024). Nevertheless, given the specific requirements and conditions associated with food‐packaging applications, dedicated LCA studies are still needed to comprehensively evaluate the environmental performance and sustainability of COF‐based food‐packaging materials.

COF production involves connecting organic molecules through covalent bonds, often requiring substantial petroleum resources, indirectly increasing greenhouse gas emissions. The synthesis process frequently requires high temperatures and significant energy consumption. Additional steps, such as washing and filtration, can also consume large amounts of flammable and toxic organic solvents like acetone and tetrahydrofuran (THF), posing safety risks. To address these challenges, researchers are exploring alternative methods for COF production, including mechanochemical techniques that eliminate the need for organic solvents, photochemical methods with milder synthesis conditions, and RT synthesis processes (Ge et al. 2024).

Owing to their porous and flexible structure, COFs can capture or convert environmental contaminants such as harmful gases, heavy‐metal ions, and organic dyes through physical or chemical adsorption. The environmental impact of this process, however, depends on the solvents and fabrication methods used. Espada et al. (2023) compared surface printing and 3D inkjet printing of RT‐COF‐1 using life‐cycle assessment, finding that monomer synthesis was the main contributor to environmental impact in both cases, with 3D printing showing a lower overall footprint (a 5%–65% reduction across impact categories) and comparing favorably to other 3D‐printable materials such as polymerizable ILs.

Traditional COFs often contain nondegradable organic ligands, which can lead to chemical residues in soil and water, posing potential harm to the environment. Incinerating COF may result in the release of harmful gases and particulate matter, contributing to air pollution and endangering ecosystems. To address these concerns, researchers are exploring the use of biodegradable organic ligands, non‐heavy metal catalysts, and environmentally friendly synthesis methods. These advancements aim to ensure that discarded COFs do not cause long‐term environmental damage. Once COFs have served their intended purpose in food packaging, they can safely decompose and return to the natural environment (Ge et al. 2024).

In addition, researchers employ physical or chemical techniques to repurpose discarded COFs as additives for low‐value materials or modify them for new functions, thereby extending their usefulness. This recycling process reduces the need for new material synthesis and lessens the environmental impact of waste materials, further promoting sustainable development and environmental protection of COF materials (Ge et al. 2024).

### 5.3 Green Precursors and Sustainable Synthesis Strategies for COF‐Based Food Packaging

Despite the significant functional advantages of COFs in food‐packaging applications, concerns regarding the environmental impact, toxicity, and economic cost of conventional synthesis routes and precursor materials remain critical challenges that must be addressed to enable their safe and sustainable industrial implementation. Traditional COF synthesis frequently relies on petroleum‐derived monomers, toxic organic solvents, metal catalysts, and energy‐intensive reaction conditions, which raise environmental, economic, and safety concerns. Commonly used solvents, such as DMF, *N*,*N*‐dimethylacetamide (DMAc), THF, mesitylene, and dioxane, are classified as hazardous due to their toxicity, volatility, and potential carcinogenic effects. Residual solvent molecules trapped within the COF pore structure may subsequently migrate into food systems, posing potential health risks. Furthermore, many conventional monomers used in COF synthesis, including aromatic dialdehydes, boronic acids, and amine‐based precursors, are synthesized through multistep petrochemical processes involving hazardous reagents and generating significant chemical waste (Chen, Li, et al. 2021; Zhang et al. 2025). These factors not only increase the environmental footprint of COF production but also contribute to high manufacturing costs, limiting their scalability and commercial viability for food‐packaging applications.

In response to these challenges, recent research efforts have increasingly focused on developing green synthesis strategies based on renewable, biodegradable, and environmentally benign precursor materials. One promising approach involves the use of bio‐derived monomers obtained from naturally occurring compounds such as lignin, tannic acid, flavonoids, amino acids, peptides, carbohydrates, and CDs. Lignin, one of the most abundant renewable polymers on Earth and a major by‐product of the paper and biofuel industries, has emerged as a particularly attractive building block for sustainable porous framework synthesis. Its aromatic structure, abundant hydroxyl and methoxy functional groups, and inherent biodegradability enable the formation of highly stable, functional porous networks suitable for gas adsorption and antimicrobial applications. Similarly, tannic acid, a naturally occurring polyphenol widely found in plants, offers multiple hydroxyl functional groups capable of participating in covalent condensation reactions, enabling the formation of environmentally friendly COF‐like structures. CDs, which are cyclic oligosaccharides derived from starch, provide another highly promising green precursor due to their biocompatibility, biodegradability, and well‐defined cavity structure. COFs synthesized from CD derivatives exhibit excellent safety profiles and have demonstrated effective adsorption of volatile compounds and contaminants, making them particularly suitable for food contact applications. Another important green synthesis strategy involves replacing toxic organic solvents with environmentally friendly alternatives such as water, ethanol, supercritical carbon dioxide, and DESs. Water‐based COF synthesis represents one of the most attractive approaches due to its complete elimination of toxic solvent exposure. Recent studies have demonstrated successful synthesis of highly crystalline COFs in aqueous environments under mild conditions, significantly reducing environmental impact. Ethanol, a renewable solvent derived from biomass fermentation, offers another low‐toxicity alternative that enables efficient COF synthesis while minimizing environmental hazards. DESs, formed by mixing biodegradable compounds such as choline chloride and glycerol, have gained increasing attention due to their low toxicity, low volatility, and high recyclability. These solvents provide excellent solubilization capability while significantly reducing hazardous waste generation. Mechanochemical synthesis represents another highly sustainable approach that eliminates the need for solvents entirely (Patial et al. 2022; Xue et al. 2022; Wang, Xu, et al. 2025; Xiong et al. 2024; Wu, Wang, et al. 2025; Shen et al. 2023; Qiu et al. 2025). This technique involves grinding precursor materials together under mechanical force, enabling rapid COF formation with minimal environmental impact and significantly reduced energy consumption.

In addition to green precursor selection and solvent replacement, energy efficiency represents another critical factor in sustainable COF production. Traditional solvothermal synthesis methods often require high temperatures (120–250°C) and long reaction times (24–72 h), resulting in high energy consumption and increased production costs. Green synthesis approaches, such as RT synthesis, MW‐assisted synthesis, and photochemical synthesis, offer significant energy savings while maintaining high material quality. MW synthesis, for example, can reduce reaction times from several days to minutes while significantly lowering energy consumption. Similarly, RT synthesis eliminates heating requirements entirely, representing one of the most energy‐efficient production methods available. Beyond synthesis considerations, the use of biodegradable and edible precursor materials offers significant advantages for improving the safety and environmental compatibility of COFs. Incorporating naturally occurring compounds such as amino acids, peptides, and carbohydrate derivatives enables the development of biodegradable COFs capable of decomposing into nontoxic products after disposal. This biodegradability significantly reduces environmental accumulation and long‐term ecological impact. Furthermore, the use of food‐grade precursor materials enhances safety for direct food contact applications and simplifies regulatory approval processes (Guo, Ma, et al. 2025; Mu et al. 2018; Cao et al. 2025).

Economic considerations also strongly favor the adoption of green precursor materials. Many bio‐derived monomers, such as lignin, starch, and cellulose derivatives, are widely available as industrial by‐products at relatively low cost. Utilizing these abundant renewable resources significantly reduces raw material costs and improves the economic feasibility of large‐scale production. In contrast, conventional synthetic monomers often require complex chemical synthesis and purification, increasing production costs and limiting scalability. The development of sustainable COFs also aligns closely with global regulatory trends and environmental policies aimed at reducing plastic waste and promoting environmentally friendly packaging materials. Regulatory agencies, such as the European Food Safety Authority (EFSA) and the United States Food and Drug Administration (FDA), increasingly emphasize the importance of using safe, nontoxic, and environmentally sustainable materials in food‐packaging applications. Green synthesis approaches based on renewable precursor materials significantly improve the likelihood of regulatory approval and facilitate commercial implementation. Furthermore, the integration of green chemistry principles into COF design enables the development of multifunctional packaging systems that combine environmental sustainability with advanced functional performance. For example, COFs synthesized from natural polyphenols exhibit inherent antimicrobial and antioxidant properties, eliminating the need for additional chemical additives. This multifunctionality further enhances safety and reduces overall chemical usage. Despite these promising advances, several challenges remain in the widespread adoption of green COF synthesis strategies. Controlling crystallinity, pore structure, and functional performance using bio‐derived precursors remains more difficult compared to conventional synthetic monomers. Additionally, achieving consistent large‐scale production while maintaining material quality requires further optimization. Nevertheless, ongoing research continues to demonstrate that green precursor materials and sustainable synthesis methods represent a highly promising pathway toward environmentally friendly COF‐based food‐packaging systems. By replacing toxic petrochemical reagents with renewable, biodegradable, and low‐cost alternatives, it is possible to significantly reduce environmental impact while improving safety and economic feasibility (Cheng et al. 2024; Li et al. 2022; Huang et al. 2025; Hu et al. 2024). These advances represent a critical step toward the development of next‐generation sustainable packaging materials capable of addressing global food preservation and environmental challenges.

### 5.4 Regulatory Barriers

The application of advanced porous materials, such as COFs and MOFs in food packaging, is an emerging field, with current efforts focused on extending shelf life, enabling active packaging, and supporting intelligent sensing (Xu et al. 2023; Zhang et al. 2024). However, the use of these materials in FCMs is subject to strict safety regulations, toxicological limitations, and migration assessments. At present, no specific international regulation explicitly targets COFs in food packaging; instead, COF‐containing packaging is governed under broader legislation covering FCMs, nanotechnology, and plastic additives. Under major regulatory frameworks, such as European Union (EU) Regulation (EC) No. 1935/2004 and the US FDA's Federal Food, Drug, and Cosmetic Act, any material intended to contact food must be manufactured in accordance with good manufacturing practices (GMP) (Zhang et al. 2024; Kato and Conte‐Junior 2021), and must not transfer constituents into food at levels that could endanger human health, cause an unacceptable change in food composition, or deteriorate its organoleptic properties. Because many COFs and MOFs operate at the nanoscale or are incorporated as nanofillers, they are additionally subject to stringent provisions governing engineered nanomaterials; EU Regulation (EU) No. 10/2011, for example, requires that substances in nanoform be explicitly authorized and risk‐assessed before use in plastic FCMs, given that their physicochemical properties can differ substantially from their bulk‐phase counterparts (Zhang et al. 2024).

Regulatory approval and practical deployment of COFs in food packaging hinge on several critical risk parameters, foremost among them the potential migration of toxic components into the food matrix. COFs are synthesized from organic linkers and building blocks such as aldehydes, amines, or phenolic compounds; when polymerization is incomplete, unreacted monomers or precursors may remain trapped within the framework and subsequently migrate into food, posing a chemical hazard. Residual solvents used during synthesis raise similar concerns and must comply with strict specific migration limits (SML) or total migration limits, for instance, 10 mg/dm^2^ of surface area under EU regulations, before packaging can be deemed safe for consumer use. A related challenge arises from non‐intentionally added substances (NIAS), which can form during the manufacturing, storage, or degradation of COF‐modified packaging. These include side‐products of the polymerization reaction, degradation products resulting from exposure to food simulants, thermal processing, or UV light, and impurities present in the starting raw materials. Identifying and performing risk assessments on these unpredictable NIAS remain a major analytical challenge for food safety authorities (Kato and Conte‐Junior 2021; Zhang et al. 2024).

Before a COF can be widely commercialized in food packaging, comprehensive toxicological profiling is also required. This includes demonstrating that the COF structure remains stable when exposed to different food types, acidic, fatty, aqueous, or alcoholic, over specified shelf life periods, as well as thoroughly evaluating, through in vitro and in vivo studies, the cellular toxicity, genotoxicity, and bioaccumulation potential of migrated COF particles or degraded linkers, which is necessary to establish acceptable daily intake (ADI) limits. Although the potential of crystalline porous frameworks like COFs and MOFs in active food packaging is substantial, their commercial implementation remains heavily constrained by the need to prove zero or negligibly low migration of toxic precursors, residues, and NIAS into food products (Kato and Conte‐Junior 2021; Zhang et al. 2024).

## 6 Performance of COF‐Based Packaging Under Practical Supply‐Chain Conditions

Safety in packaging design for food products generally refers both to preserving a high quality level and to preventing damage to the package and its contents during handling, transportation, and other logistic processes (Manzini and Accorsi 2013). Although systematic evaluation of COF‐based packaging throughout the entire food supply chain remains limited, recent studies have investigated its performance under environmentally relevant conditions, including temperature variation, high relative humidity, and mechanical stress, which are critical factors during transportation and storage. Han et al. (2023) developed a silk‐fibroin/2D‐COF packaging system specifically for climate‐stressed environments and evaluated its performance under approximately 35°C and 75% relative humidity. Under these conditions, the COF‐containing packaging, particularly the hexanal‐loaded system, substantially suppressed mold growth in packaged soybeans, achieving a reduction of approximately four orders of magnitude compared with polyethylene cling film, demonstrating its potential for food preservation under warm and humid storage conditions. In another study, sodium alginate/COF‐based films containing citral were evaluated for active food packaging and showed antimicrobial activity and mold‐inhibitory performance under conditions associated with elevated temperature and humidity, including applications to poultry and peanuts (Singh et al. 2024).

Temperature fluctuations during storage and transport have also been addressed through the development of COF‐based intelligent packaging systems with time–temperature‐responsive behavior. A recent study incorporated a responsive COF into a chitosan/PVA matrix and demonstrated temperature‐dependent color changes across a broad range (−22°C, 4°C, 25°C, and 55°C), enabling monitoring of temperature history during storage and transportation while also contributing to food preservation (Han et al. 2023). Beyond these environmental stresses, the incorporation of COFs can influence the mechanical integrity of packaging films, which is important for withstanding handling and transportation. Studies of HPMC/TZP‐COF films and chitosan films containing COF‐Ag NPs have reported enhanced tensile strength or stiffness resulting from improved interfacial interactions, network compactness, and reinforcement by the COF phase (García‐Arroyo et al. 2020; Dai et al. 2022); however, excessive COF loading or particle aggregation may introduce stress‐concentration sites and compromise flexibility. Collectively, these findings indicate that COF‐based packaging has promising potential to maintain functionality under variable temperature and humidity conditions while providing mechanical reinforcement, although comprehensive validation under real‐world transportation and supply‐chain scenarios remains an important area for future research.

## 7 Role of AI and Machine Learning (ML) in Advancing COF‐Based Food Packaging

The AI and ML approaches into the development of COFs for food packaging represent a transformative opportunity to accelerate innovation, optimize material performance, and address key challenges in scalability and application‐specific design. This convergence of computational intelligence with materials science is creating new paradigms for rational material design that overcome traditional trial‐and‐error experimental approaches.

### 7.1 Accelerating COF Discovery and Design

The vast chemical space available for COF design, encompassing numerous building blocks, linker combinations, and synthetic pathways, presents both an opportunity and a challenge. Traditional experimental approaches can explore only a small fraction of possible structures, whereas AI‐driven methods can rapidly screen millions of hypothetical frameworks. Recent advances in generative models, particularly transformer‐based architectures and graph neural networks (GNNs), have demonstrated remarkable capability in navigating this complex structural landscape (Korolev and Mitrofanov 2024). These approaches enable the generation of novel COF structures with optimized pore geometries specifically tailored for the selective adsorption of food‐relevant volatile organic compounds. Similarly, reinforcement learning approaches can design COFs with enhanced thermal stability through the identification of novel linkage patterns that maximize framework rigidity while maintaining high surface area. The prediction of COF properties represents another crucial area where ML approaches demonstrate significant advantages. Traditional density functional theory (DFT) calculations, while accurate, are computationally intensive and impractical for high‐throughput screening. ML models trained on experimental and computational datasets can predict key properties, including surface area, pore volume, thermal stability, and adsorption selectivity, with remarkable accuracy and at a fraction of the computational cost. These predictive capabilities are particularly valuable for food‐packaging applications, where multiple properties must be simultaneously optimized to achieve desired functionality. Table 3 outlines next‐generation AI/ML platforms for COF discovery and development projected for the period 2025–2035.

**TABLE 3:** Next‐generation artificial intelligence (AI)/machine learning (ML) platforms for covalent organic framework (COF) discovery and development (2025–2035).

Platform type	Key capabilities	Technical foundation	Projected impact on food packaging	Timeline to implementation
Autonomous materials discovery systems	• Self‐directed experimentation with zero human intervention • Closed‐loop learning across synthesis, characterization, and testing • Predictive modeling with <1% error for 20+ properties simultaneously	• Quantum‐classical hybrid algorithms • Self‐improving synthetic robotics • Real‐time interpretable ML models	• 100× acceleration in discovery‐to‐application pipeline • COFs with designer response to specific food degradation markers • Complete elimination of food waste from packaging failure	5–8 years for partial implementation; 10–15 years for full deployment
Cognitive Synthesis Engineers	• Natural language processing of entire materials science literature • Intuitive reasoning about chemical reactivity • Identification of non‐obvious synthetic shortcuts • Cross‐disciplinary knowledge transfer	• Foundation models trained on comprehensive chemical knowledge • Causal inference engines • Multi‐agent collaborative systems	• Green synthesis routes using only food‐grade precursors • Room‐temperature, water‐based COF synthesis protocols • COF production costs reduced by 95% through process innovation	3–5 years for basic assistance; 7–10 years for expert‐level capabilities
Quantum‐enhanced design systems	• Exact property prediction from first principles at negligible computational cost • Design of quantum‐effect enhanced properties (coherence, entanglement) • Navigation of high‐dimensional design spaces (100+ parameters)	• Quantum machine learning algorithms • Fault‐tolerant quantum computers • Quantum‐classical neural networks	• Revolutionary barrier properties from quantum coherence effects • Atomically precise COF structures with zero defects • Complete elimination of oxygen permeability	8–12 years for initial applications; 15–20 years for transformative capabilities
Molecular digital twins	• Perfect digital replicas of COF behavior under all conditions • Real‐time prediction of COF‐food interactions • Simulation of entire product lifecycle, including degradation	• Exascale computing systems • Multi‐physics simulation engines • Adaptive resolution modeling	• Virtual testing replacing 99% of physical experiments • Precise shelf life prediction for any food‐COF combination • Preemptive identification of all possible failure modes	5–7 years for basic twins; 10–12 years for comprehensive models
Neuro‐inspired design networks		• Artificial general intelligence modules • Neural architecture search • Bio‐inspired algorithms	• COF structures mimicking plant cuticle functionality • Self‐healing packaging based on neural‐inspired adaptation mechanisms • Emergence of entirely new property combinations	7–10 years for initial systems; 15+ years for advanced capabilities

COFs have emerged as highly versatile materials, not only for biosensing but also as prospective functional components in advanced food‐packaging systems. Their tunable chemical and electronic structures enable the development of chromatic sensors capable of detecting subtle environmental or chemical changes, a property that can be directly translated to monitoring food quality and safety (Han et al. 2023; Hu et al. 2024). For instance, COFs integrated within biocompatible matrices, analogous to SF microneedles in plant systems, could be engineered as smart packaging layers to detect pH shifts, volatile organic compounds, or microbial metabolites, providing real‐time, noninvasive indicators of spoilage or contamination (Wang et al. 2024). By exploiting the 3D spatial mapping capability demonstrated in chemical tomography applications, COF‐based films or coatings could offer multidimensional monitoring of the food matrix, revealing heterogeneity in freshness or chemical degradation that traditional single‐point sensors cannot capture. The modularity of COFs, including adjustable pore sizes, electron donor–acceptor characteristics, and tunable proton affinities, offers a powerful toolkit for designing packaging materials that combine mechanical stability, selective sensing, and barrier functionality (Figure 8a–i) (Geng et al. 2020; Huang et al. 2016). Nanoporous COFs can rapidly respond to changes in the local chemical environment, whereas their insoluble polymeric framework minimizes leaching and contamination, crucial considerations for direct food contact applications. By selecting monomers with biocompatible and food‐safe components, such as amino acids, CDs, or naturally derived aldehydes, future COF‐based packaging can be designed to maximize both performance and safety, while providing multifunctionality, such as spoilage detection, freshness visualization, and controlled release of preservatives or antioxidants. In perspective, integrating chromatic COF sensors into packaging aligns with the broader goals of smart and sustainable food systems. Coupled with AI‐guided design, COFs could be rapidly screened and optimized for selective adsorption of target analytes, thermal and chemical stability, and synergistic barrier properties when combined with polymers. Such AI‐informed approaches will accelerate the development of next‐generation food‐packaging materials capable of dynamic sensing and protection, providing a transformative tool to ensure product quality, enhance shelf life, and reduce food waste while maintaining consumer safety.

**FIGURE 8:**
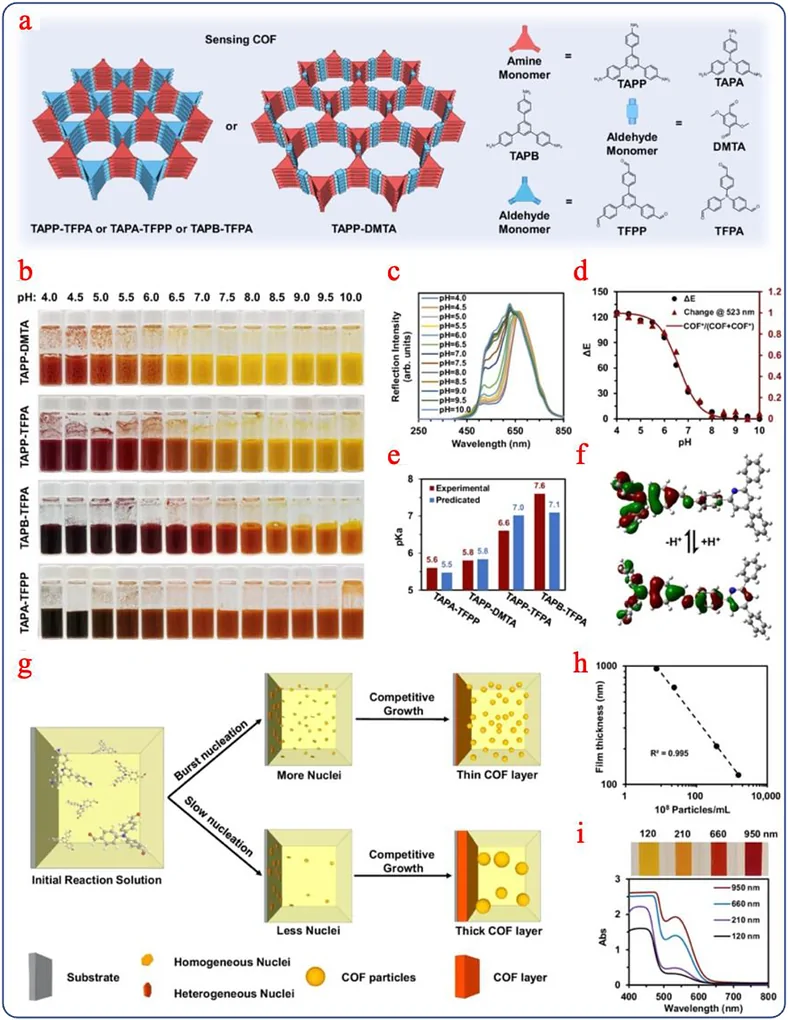
(a) Chemical structures of the covalent organic frameworks TAPP–TFPA, TAPA–TFPA, TAPB–TFPP, and TAPP–DMTA alongside their corresponding monomeric units. (b) Photographs showing the dispersion behavior of TAPP–DMTA, TAPP–TFPA, TAPB–TFPA, and TAPA–TFPP powders in buffer solutions across a range of pH values, illustrating pH‐dependent solubility and aggregation. (c) Reflectance spectra of TAPP–TFPA powders exposed to buffers of varying pH, demonstrating shifts in optical response as a function of protonation state. (d) Quantitative analysis of the energy change (Δ*E*) and relative reflectance at 523 nm for TAPP–TFPA powders across the pH gradient, fitted to the Henderson–Hasselbalch equation to reveal an effective p*K*a of 6.6. (e) Comparison of experimentally determined p*K*a values with predicted p*K*a calculated from simulated proton affinities in aqueous media for the various COFs. (f) Density functional theory (DFT) optimized geometries and the highest occupied molecular orbitals (HOMOs) of the TAPP–TFPA repeat unit in both unprotonated and protonated states, illustrating electronic structure modulation upon protonation. (g) Schematic representation of the homogeneous nucleation limitation strategy employed during film formation. (h) Correlation between film thickness and particle density for TAPP–TFPA, indicating control over morphological properties. (i) Visual images and UV‐Vis absorption spectra of TAPP–TFPA films of varying thickness on PET substrates following treatment with a pH 5.0 buffer, highlighting the effect of film thickness on optical response (Wang et al. 2024). This article is licensed under a Creative Commons Attribution 4.0 International License.

### 7.2 Optimizing Synthesis and Processing

The translation of computationally designed COFs into experimentally realizable materials remains a significant challenge, with synthesis conditions often requiring extensive optimization. ML approaches are increasingly addressing this synthesis gap through the development of reaction condition prediction models and autonomous experimentation platforms. Bayesian optimization frameworks can reduce the number of experiments required to achieve high‐crystallinity COFs, improving crystallinity by up to ≈80% in a single optimization round, and can identify nonintuitive solvent combinations and catalyst concentrations that maximize framework order while minimizing reaction time (Shao et al. 2025). This approach is particularly valuable for scaling up COF production, a critical consideration for commercial food‐packaging applications where material costs and production efficiency are paramount. Computer vision algorithms integrated with automated synthesis platforms demonstrate another powerful approach for synthesis optimization. These systems can continuously monitor crystallization processes through in situ spectroscopic techniques and adjust reaction parameters in real‐time to maximize yield and product quality. Advanced autonomous synthesis platforms can employ convolutional neural networks (CNNs) to analyze powder x‐ray diffraction patterns during COF formation, achieving high success rates in synthesizing highly crystalline materials across diverse structural libraries. For food‐packaging applications, where material homogeneity and batch‐to‐batch consistency are essential for regulatory approval, such AI‐driven synthesis optimization represents a transformative capability. Post‐synthetic modification of COFs, crucial for introducing specific functional groups for food‐packaging applications, similarly benefits from ML‐guided approaches. Reinforcement learning frameworks can efficiently navigate the complex parameter space of reaction conditions, reagent concentrations, and processing sequences to maximize functionalization efficiency while preserving framework integrity. ML‐optimized protocols for antimicrobial peptide conjugation to imine‐linked COFs can achieve significantly higher loading capacity than conventional methods while maintaining framework crystallinity and porosity, properties essential for effective antimicrobial food‐packaging applications.

### 7.3 Structure–Property–Performance Relationships for Food‐Packaging Applications

The complex relationships between COF molecular structure, bulk material properties, and functional performance in food‐packaging applications are ideally suited for exploration through ML approaches. Deep learning models can identify nonlinear, multi‐parameter correlations that elude traditional structure–property analyses. Hierarchical ML frameworks that integrate molecular descriptors, synthetic parameters, and material properties can predict oxygen and water vapor transmission rates for COF–polymer composite films. These models can reveal unexpected synergistic effects between COF pore geometry and polymer chain configuration that significantly enhance barrier performance beyond what either component could achieve independently, insights that lead to the development of high‐performance films with oxygen permeability reduced by 87% compared to conventional packaging materials. For active packaging applications, where COFs function as carriers for antimicrobial or antioxidant compounds, ML models can optimize release kinetics by establishing correlations between framework structure, guest molecule properties, and environmental triggers. Recurrent neural network models can accurately predict the temperature‐dependent release profiles of antimicrobial compounds from structurally diverse COFs. These models enable the design of COF‐based active packaging systems with precisely controlled release behavior, accelerating at elevated temperatures where microbial growth is favored and maintaining minimal release under refrigeration conditions. Smart packaging applications, where COFs serve as sensors for monitoring food freshness or detecting contaminants, represent another domain where ML approaches offer considerable advantages. Random forest algorithms and support vector machines have demonstrated remarkable capability in processing multi‐dimensional sensor response data from COF‐based colorimetric or fluorescent sensors, enabling accurate classification of food freshness states or contaminant levels. Smartphone‐based analysis systems employing CNNs can achieve high accuracy in classifying food freshness levels based on the colorimetric response of COF sensor arrays to volatile compounds, a significant improvement over traditional single‐threshold chemical indicator.

Beyond barrier and sensing functionalities, COFs offer substantial potential for addressing chemical contaminants in food systems, such as PFASs. The intrinsic modularity of COF frameworks, including tunable pore sizes, surface chemistry, and functional group placement, makes them ideal candidates for selective adsorption of trace pollutants. Recent studies have demonstrated that cationic‐fluorinated COFs (CF‐COFs) can be engineered as high‐performance adsorbents, exploiting synergistic interactions between quaternary ammonium sites and fluorinated guest molecules to achieve strong binding and efficient extraction (Gong et al. 2024). Molecular simulations and DFT analyses have revealed that both electrostatic attraction and fluorophilic affinity drive PFAS adsorption, providing mechanistic insights that guide rational design (Figure 9). Integrating ML approaches with such computational and experimental datasets offers a transformative avenue for predictive COF design. By correlating framework descriptors, such as pore topology, functional group density, and surface charge, with adsorption capacity and selectivity, deep learning models can identify nonlinear structure–property–performance relationships that conventional analyses may overlook. This enables rapid screening and optimization of COFs for targeted contaminant removal, facilitating the development of multifunctional food‐packaging materials that not only extend shelf life but also actively mitigate chemical hazards. Such data‐driven strategies accelerate the translation of COF‐based materials from laboratory synthesis to practical food safety applications, while providing a framework for designing future active and smart packaging systems.

**FIGURE 9:**
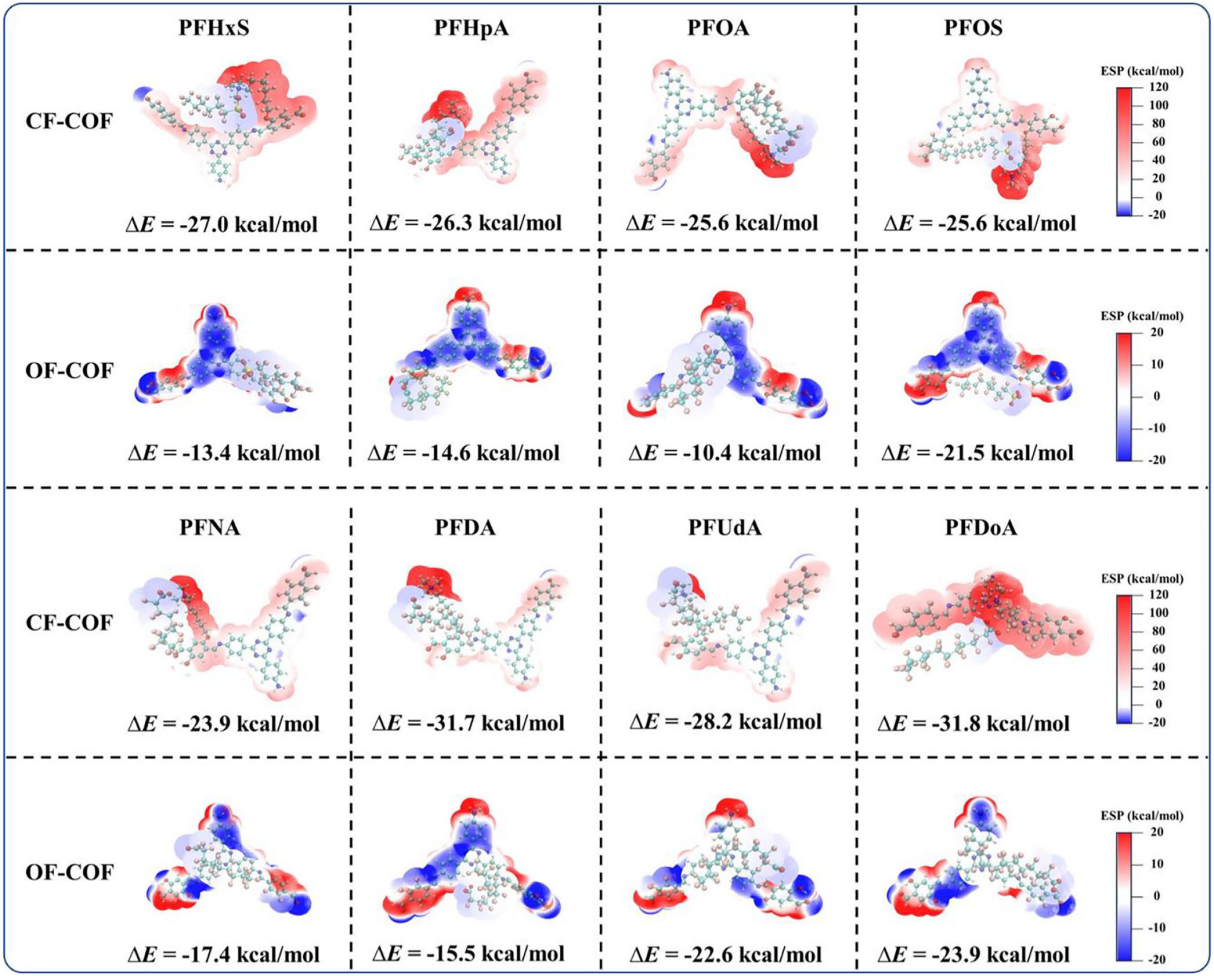
Interaction energies and electron density distributions between PFAS molecules and CF‐COF (OF‐COF) frameworks (Gong et al. 2024). Reprinted with permission from Gong et al. (2024), Copyright 2024, Elsevier. CF: cationic‐fluorinated; COF: covalent organic framework.

### 7.4 Predictive Modeling of COF‐Food Interactions

The interactions between COFs and food components represent a complex domain where molecular dynamics simulations coupled with ML approaches can provide crucial insights. These interactions are central to ensuring both the effectiveness and safety of COF‐based packaging materials. Advanced ML models, particularly physics‐informed neural networks, can predict the adsorption and potential migration of small molecules at the COF‐food interface under various temperature, humidity, and pH conditions. Hybrid quantum mechanics/molecular mechanics approaches enhanced by ML potentials can accurately model the interactions between various COF frameworks and common food additives and contaminants. These studies identify specific structural motifs that maximize adsorption selectivity for harmful migrants while minimizing interaction with beneficial food components, insights directly applicable to designing safer FCMs. Table 4 presents AI‐enabled transformative applications of COF‐based food‐packaging systems.

**TABLE 4:** AI‐enabled transformative applications for covalent organic framework (COF)‐based food‐packaging systems (2025–2040).

Application paradigm	AI‐driven capabilities	Consumer benefits	Food system transformation	Anticipated breakthrough timeline
Cognitive food guardians	• Real‐time monitoring of 500+ food quality parameters • Anticipative detection of spoilage 48–72 h before conventional methods • Self‐adapting detection thresholds based on food type • Multimodal sensing (visual, olfactory, chemical, and structural)	• Near‐zero food waste from spoilage • Dynamic shelf life information • Personalized freshness indicators • Elimination of foodborne illness	• Complete reimagining of cold chain management • Transformation of inventory systems • Elimination of “best by” dates • Revolution in food safety protocols	Initial systems: 3–5 years Advanced systems: 7–10 years Comprehensive deployment: 12–15 years
Responsive preservation networks	• Spatially resolved active agent release • Coordinated multi‐agent release sequences • Predictive response to emerging microbial threats • Biomimetic adaptation to environmental fluctuations	• Extended shelf life without preservatives • Enhanced nutrient retention • Protection tailored to specific threats • Minimal intervention preservation	• Enablement of ultra‐long‐distance food transport • Preservation of indigenous food systems • Reduction in antimicrobial resistance • New paradigms in minimal processing	Basic functionality: 2–4 years Advanced coordination: 6–8 years Autonomous systems: 10–12 years
Environmental condition optimizers	• Real‐time modification of internal package atmosphere • Humidity regulation with nanogram precision • Light filtration with wavelength specificity • Thermal management through AI‐controlled phase changes	• Perfect ripening of fruit on demand • Texture preserved at optimal state • Color and appearance maintenance • Temperature abuse protection	• Elimination of controlled atmosphere facilities • Revolution in last‐mile delivery • Transformation of consumer storage practices • New approaches to food architecture	First generation: 3–5 years Advanced systems: 8–10 years Fully integrated systems: 12–15 years
Circular material orchestrators	• Programmable end‐of‐life behavior • Self‐sorting for recycling streams • Triggered biodegradation under specific conditions • Closed‐loop material cycles with zero leakage	• Zero‐waste packaging solutions • Elimination of sorting confusion • Environmentally harmonious disposal • Enhanced sustainability credentials	• Complete redesign of recycling infrastructure • Elimination of packaging waste streams • New models for material ownership • Regenerative packaging ecosystems	Initial implementations: 4–6 years Advanced functionality: 8–12 years Complete systems: 15–20 years
Food‐package symbiosis systems	• Bidirectional communication between food and packaging • Nutrient exchange and preservation • Packaging that evolves with food state • Bioactive interface management	• Enhanced nutritional value • Novel sensory experiences • Extended functionality beyond protection • Minimal separation between packaging and food	• Blur between food and packaging • New food product categories • Revolution in culinary techniques • Transformation of food manufacturing	Conceptual demonstrations: 5–7 years Functional prototypes: 10–12 years Commercial systems: 15–20 years
Ethical consumption advisors	• Supply‐chain transparency verification • Environmental impact quantification • Allergen and sensitivity monitoring • Personalized nutrition guidance	• Perfect alignment with personal ethics • Confidence in product provenance • Safety for vulnerable individuals • Optimized nutritional intake	• Transformation of consumer trust models • Revolution in food marketing • New paradigms in personalized nutrition • Evolution of dietary practice	Basic information: 2–4 years Advanced guidance: 5–8 years Comprehensive systems: 10–15 years

For regulatory approval of novel food‐packaging materials, comprehensive toxicological assessment is essential. ML approaches are increasingly valuable in this domain, enabling in silico prediction of potential toxicity risks and guiding experimental studies. Transformers trained on toxicological datasets can predict potential degradation products of COFs under various conditions and assess their biocompatibility. Graph attention networks can predict the cytotoxicity of COF degradation products with high accuracy across multiple cell lines, significantly accelerating the safety assessment process for novel framework structures. These ML‐guided toxicological evaluations are particularly valuable given the stringent safety requirements for FCMs and the need to minimize animal testing.

### 7.5 Integration of AI in Manufacturing and Quality Control

Beyond material discovery and optimization, AI approaches are transforming the manufacturing and quality control processes for COF‐based packaging materials. Computer vision systems integrated with manufacturing lines can perform real‐time defect detection and quality assessment of COF–polymer composite films with accuracy exceeding human inspection capabilities. Deep learning systems for inline monitoring of COF distribution within polymer matrices during film extrusion can achieve near‐perfect detection accuracy for aggregation defects as small as 5 µm, critical for ensuring consistent barrier and mechanical properties in the final packaging material. Digital twins, AI‐powered virtual replicas of physical manufacturing processes, enable predictive maintenance and process optimization for COF production facilities. These systems continuously analyze sensor data from synthesis reactors, functionalization chambers, and film formation equipment to identify optimal operating conditions and predict potential failures before they occur. Implementation of digital twin systems for COF membrane production can reduce energy consumption by 20%–25% and material waste by 35%–40% while improving product consistency, advances with significant implications for the commercial viability and sustainability of COF‐based packaging materials.

Despite the transformative potential of AI and ML in advancing COF‐based food packaging, several challenges must be addressed to fully realize this potential. Data scarcity and quality represent fundamental limitations, with experimental datasets often being sparse, heterogeneous, and collected under inconsistent conditions. Federated learning approaches that enable collaborative model training across multiple research institutions while preserving data privacy show promise for addressing these limitations. Additionally, the development of standardized data reporting formats and automated extraction tools for COF literature could significantly enhance the quality and quantity of training data available for ML models. Interpretability of ML models remains another crucial challenge, particularly for regulatory approval processes where transparent reasoning is essential. The development of explainable AI frameworks specifically tailored to materials science applications is an active research area with considerable relevance for COF development. Attention‐based architectures that highlight structure–property relationships and visualize which molecular features most strongly influence predicted properties represent a promising direction for enhancing model interpretability. Looking forward, the integration of multi‐scale modeling approaches, spanning quantum mechanical calculations, mesoscale simulations, and continuum modeling, with hierarchical ML architectures, presents perhaps the most promising frontier for COF design. Such integrated approaches could enable seamless prediction across length and time scales, connecting molecular structure to macroscopic performance in complex food‐packaging environments. Early implementations of such multi‐scale ML frameworks can accurately predict oxygen permeability, mechanical durability, and antimicrobial efficacy for COF–polymer composites under simulated food storage conditions, capabilities essential for designing application‐specific packaging solutions. Active learning frameworks that continuously integrate experimental feedback to refine model predictions represent another promising direction. These systems could eventually enable fully autonomous discovery platforms where algorithms design experiments, interpret results, and iteratively refine COF structures to optimize performance for specific food‐packaging applications. The convergence of robotics, high‐throughput characterization, and ML‐driven experimental design could potentially accelerate COF development cycles by orders of magnitude, transforming the traditionally slow materials development timeline.

## 8 Future Perspective

The integration of COFs into food‐packaging systems represents a promising frontier at the intersection of materials science, food technology, and sustainability. Figure 10 provides a comprehensive analysis of the future landscape for COF‐based food‐packaging materials. When evaluating the sustainability profile of packaging materials (Figure 10a), COF‐based systems demonstrate notable advantages over both traditional plastics and current‐generation bioplastics. Although bioplastics excel in biodegradability (0.8 vs. 0.6 for COFs), COF‐based materials show superior performance in functional effectiveness (0.95), material safety (0.9), recyclability (0.9), and reduced carbon footprint (0.8). This balanced sustainability profile positions COFs as promising candidates for addressing the multifaceted challenges of next‐generation packaging that must simultaneously meet performance requirements while minimizing environmental impact. The moderate biodegradability score of COFs (0.6) highlights an area where further research, particularly in designing COFs with cleavable linkages that respond to specific environmental conditions, could yield significant advances.

**FIGURE 10:**
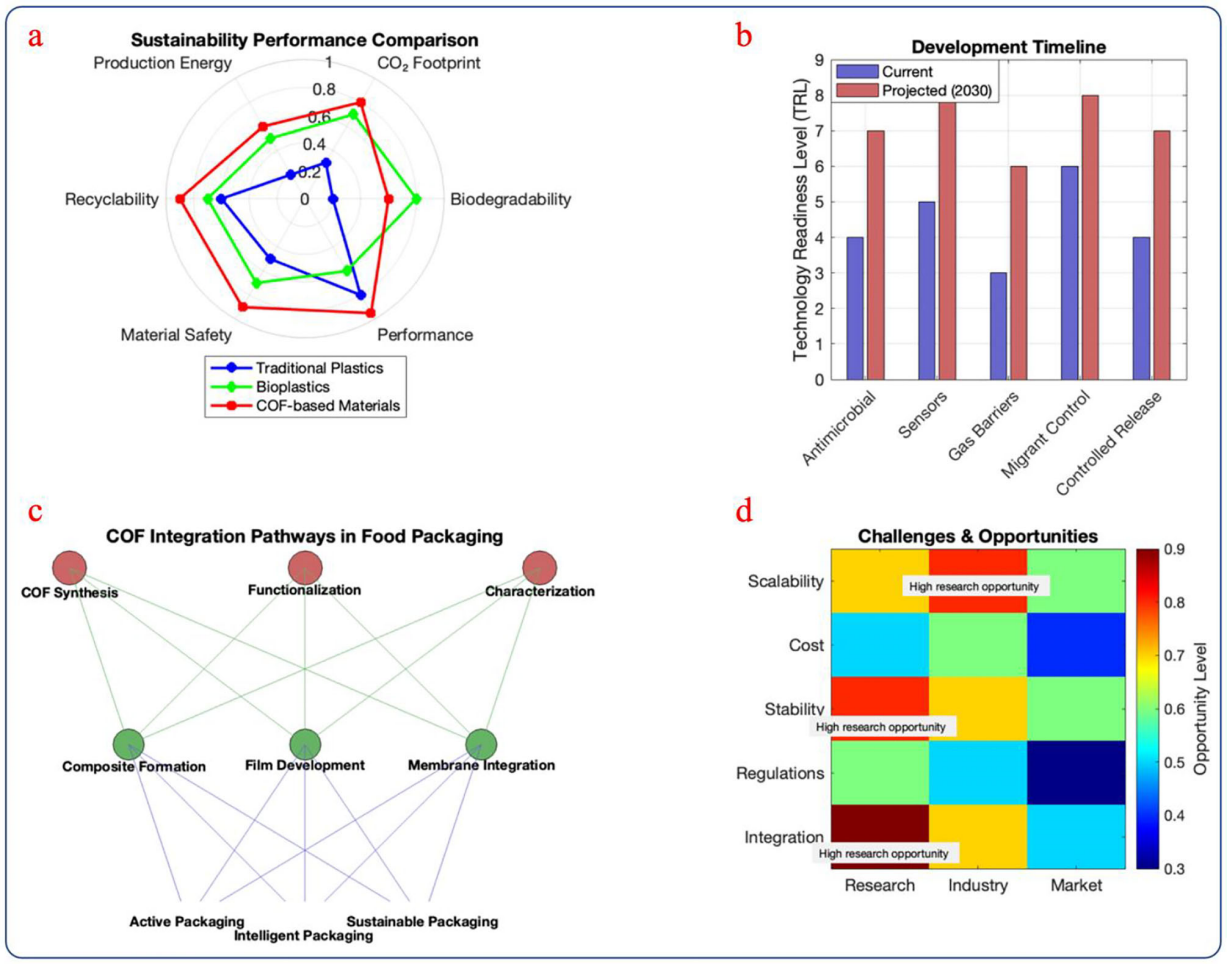
Comprehensive analysis of future prospects, challenges, and integration pathways for covalent organic frameworks (COFs) in food‐packaging applications. (a) Sustainability radar chart comparing COF‐based materials against traditional plastics and bioplastics across six key environmental and performance metrics (scale: 0–1, higher values indicate superior performance). COF‐based materials demonstrate exceptional functional performance (0.95) and material safety (0.9) while maintaining competitive advantages in recyclability (0.9) and carbon footprint reduction (0.8). (b) Technology readiness level (TRL) assessment of five primary COF applications in food packaging, showing current status (blue) and projected development by 2030 (red). Migrant control applications currently demonstrate the highest maturity (TRL 6), while sensing and migrant control technologies are projected to reach near‐commercial readiness (TRL 8) by 2030. (c) Integration pathway network visualizing the developmental routes from fundamental COF research (red nodes) through processing stages (green nodes) to final applications (blue nodes). Multiple interconnected pathways illustrate the transdisciplinary nature of successful implementation, with advances in fundamental chemistry potentially catalyzing progress across multiple application domains. (d) Challenge‐opportunity matrix depicting the relationship between five key implementation barriers and their corresponding opportunity levels across research, industry, and market domains. Integration methods at the research level (0.9) and scalability improvements at the industry level (0.8) present the highest strategic opportunities for advancing COF‐based packaging solutions. This multifaceted analysis provides a roadmap for prioritizing research efforts and highlights the potential of COFs to address critical challenges in sustainable food packaging. COF: covalent organic framework.

The technology readiness assessment (TRL, Figure 10b) evaluates the maturity rate of the technology of different COF applications in food packaging. Notably, migrant adsorption applications have reached the highest current TRL, indicating successful demonstration in relevant environments, whereas gas barrier applications remain at an earlier stage, still requiring proof‐of‐concept validation. Projections to 2030 suggest that sensing and migrant control applications will increase (system completion and qualification), positioning them for early commercial adoption. This differential in development trajectories necessitates application‐specific research strategies, with more fundamental studies needed for gas barrier applications, while sensing and migrant control technologies may benefit from focusing on scale‐up and system integration challenges. The integration pathways illustrated in Figure 10c highlight the interconnected nature of COF development from fundamental synthesis to practical applications. Multiple developmental routes are evident, with each foundational aspect (synthesis, functionalization, and characterization) influencing various intermediate processing stages and ultimately determining the functional properties in final packaging applications. This network visualization emphasizes that advances in fundamental COF chemistry, particularly in developing scalable, RT synthesis methods, can catalyze progress across multiple application domains simultaneously. The convergence of multiple pathways toward smart packaging applications underscores the synergistic potential of combining sensing, controlled release, and barrier properties within integrated COF‐based systems.

The successful translation of COF‐based packaging from laboratory to commercial applications will require coordinated efforts across multiple domains: development of scalable, environmentally benign synthesis methods; comprehensive safety assessments addressing both acute and chronic exposure scenarios; establishment of regulatory frameworks for novel FCMs; and demonstration of economic viability relative to conventional packaging solutions. Future safety research should adopt a systematic framework encompassing migration, chronic toxicity, and environmental fate. Migration studies should quantify the release of COFs, residual monomers, degradation products, and active components into relevant food simulants under realistic temperature and storage conditions. Long‐term toxicological assessments should move beyond acute cytotoxicity toward repeated‐exposure studies based on realistic dietary exposure scenarios. In parallel, environmental‐fate studies should evaluate the degradation, persistence, transformation products, and ecotoxicity of COFs under relevant soil, compost, freshwater, and marine conditions. Standardized reporting of COF structure, particle characteristics, loading level, exposure conditions, and analytical methods would also improve comparability among studies and facilitate future regulatory risk assessment.

Public acceptance and societal considerations represent an additional challenge for the future commercialization of COF‐based food‐packaging materials. Although direct consumer‐acceptance studies on COF‐based packaging are currently limited, research on nanotechnology in food packaging indicates that consumer acceptance is influenced by perceived health and environmental risks, trust in regulatory oversight, and perceived benefits (Ahmad et al. 2024). Potential concerns include migration of COFs or their degradation products into food, uncertainties regarding long‐term toxicity, and environmental persistence (Priyadarshi et al. 2026). Therefore, comprehensive migration, toxicological, and environmental‐safety assessments, together with transparent communication and appropriate regulatory frameworks, will be important for building consumer confidence in COF‐based packaging.

An additional priority is the transition from laboratory‐scale demonstrations to application‐relevant and pilot‐scale validation. Most reported COF studies remain at the milligram scale, whereas practical implementation requires reproducible large‐scale synthesis, cost‐effective processing, and conversion of COF powders into application‐ready forms such as films and membranes. Recent advances in flux and other solvent‐reduced synthesis methods have demonstrated the feasibility of kilogram‐ to ton‐scale COF production. Notably, kilogram‐scale production of selected COFs has been achieved at reported costs of less than approximately $50 kg^−1^ for NKCOF‐41 and approximately $69 kg^−1^ for NKCOF‐62; however, these values are specific to particular COF systems and should not be considered representative of the COF family as a whole. The economic viability and reproducibility of large‐scale production remain strongly dependent on the COF structure, monomer availability, synthesis route, and processing requirements. Moreover, successful commercialization requires careful consideration of production cost, safety, space–time yield, sustainability, reproducibility, and service life, together with prototype and pilot‐scale testing under realistic operating conditions. Therefore, future research should move beyond material‐level performance toward scalable synthesis, application‐specific shaping, batch‐to‐batch consistency, techno‐economic and sustainability assessment, and prototype validation under realistic conditions to facilitate the translation of COF‐based packaging toward industrial applications (Wang, Qiao, et al. 2025).

The integration of AI and ML approaches offers unprecedented opportunities to accelerate this translation by enabling rapid screening of COF structures, optimization of synthesis conditions, and prediction of long‐term performance under real‐world conditions. The challenge‐opportunity matrix (Figure 10d) provides strategic guidance for prioritizing research and development efforts. Integration methods present the highest opportunity at the research level (0.9), suggesting that innovative approaches for incorporating COFs into existing packaging materials and manufacturing processes could yield significant near‐term advances. Concurrently, scalability improvements show substantial opportunity at the industry level, reflecting the critical importance of transitioning from laboratory‐scale synthesis to commercially viable production volumes. The gradually decreasing opportunity levels from research to market across all challenges reflect the typical technology commercialization journey and highlight the importance of academic‐industrial partnerships to bridge these translational gaps. Looking forward, several critical directions emerge for advancing COF applications in food packaging: (1) development of green synthesis routes employing environmentally benign solvents, catalysts, and reaction conditions; (2) comprehensive toxicological assessment of COF materials and their degradation products; (3) engineering of stimuli‐responsive COF systems capable of adapting to fluctuating conditions throughout the food supply chain; and (4) integration of artificial intelligence and ML approaches to accelerate the discovery and optimization of application‐specific COF structures. The intersection of these research directions with emerging regulatory frameworks for novel FCMs will ultimately determine the trajectory and impact of COF‐based technologies in addressing the global challenges of food preservation, safety, and waste reduction. COFs offer exceptional potential for revolutionizing food packaging through their unique combination of structural tunability, functional versatility, and promising sustainability characteristics. Although significant challenges remain, particularly in scalability, cost‐effectiveness, and regulatory approval, the strategic research opportunities identified in this analysis provide a clear roadmap toward realizing the transformative potential of these advanced materials in creating more sustainable and effective food‐packaging systems.

## 9 Conclusion

COFs represent a transformative class of porous materials with exceptional potential to redefine next‐generation food‐packaging systems. Their metal‐free composition, tunable pore architecture, high surface area, and chemical stability enable multifunctional performance, including active preservation, antimicrobial action, and real‐time food freshness monitoring. Compared with conventional porous materials, COFs offer superior structural precision, biocompatibility, and functional versatility, positioning them as ideal candidates for smart packaging applications. Despite these advantages, challenges related to large‐scale synthesis, long‐term safety evaluation, regulatory approval, and sustainable production remain critical barriers to industrial adoption. Emerging advances in green synthesis, biodegradable COF design, and AI‐assisted material discovery are expected to accelerate the transition of COFs from laboratory research to commercial deployment. COFs provide a powerful platform for developing intelligent, sustainable, and high‐performance packaging technologies capable of significantly reducing food waste, enhancing food safety, and supporting global sustainability goals.

## Author Contributions

**Sara Esteghlal**: conceptualization, writing – original draft, writing – review and editing, visualization. **Hossein Daneshgar**: conceptualization, writing – original draft, writing – review and editing, visualization. **Navid Rabiee**: conceptualization, writing – original draft, writing – review and editing, project administration, supervision. **Mohammadreza Khalesi**: writing – review and editing, writing – original draft, conceptualization, project administration, supervision.

## Funding

The authors have nothing to report.

## Conflicts of Interest

The authors declare no conflicts of interest.
